# The recent advance and prospect of natural source compounds for the treatment of heart failure

**DOI:** 10.1016/j.heliyon.2024.e27110

**Published:** 2024-02-25

**Authors:** Xing-Juan Chen, Si-Yuan Liu, Si-Ming Li, Ji-Kang Feng, Ying Hu, Xiao-Zhen Cheng, Cheng-Zhi Hou, Yun Xu, Mu Hu, Ling Feng, Lu Xiao

**Affiliations:** a China Academy of Chinese Medical Sciences Guang’anmen Hospital, Beijing, 100053, China; b Beijing University of Chinese Medicine, Beijing, 100029, China; c Linyi Zhengzhi Hospital, Linyi, 276004, China; d First Teaching Hospital of Tianjin University of Traditional Chinese Medicine, National Clinical Research Center for Chinese Medicine Acupuncture and Moxibustion, Tianjin, 300381, China; e Peking University International Hospital, Beijing, 102206, China

**Keywords:** Heart failure, Natural product, Myocardial damage, Myocardial remodeling, Clinical treatment

## Abstract

Heart failure is a continuously developing syndrome of cardiac insufficiency caused by diseases, which becomes a major disease endangering human health as well as one of the main causes of death in patients with cardiovascular diseases. The occurrence of heart failure is related to hemodynamic abnormalities, neuroendocrine hormones, myocardial damage, myocardial remodeling *etc*, lead to the clinical manifestations including dyspnea, fatigue and fluid retention with complex pathophysiological mechanisms. Currently available drugs such as cardiac glycoside, diuretic, angiotensin-converting enzyme inhibitor, vasodilator and β receptor blocker *etc* are widely used for the treatment of heart failure. In particular, natural products and related active ingredients have the characteristics of mild efficacy, low toxicity, multi-target comprehensive efficacy, and have obvious advantages in restoring cardiac function, reducing energy disorder and improving quality of life. In this review, we mainly focus on the recent advance including mechanisms and active ingredients of natural products for the treatment of heart failure, which will provide the inspiration for the development of more potent clinical drugs against heart failure.

## 1 Introduction

**Fig. 1:**
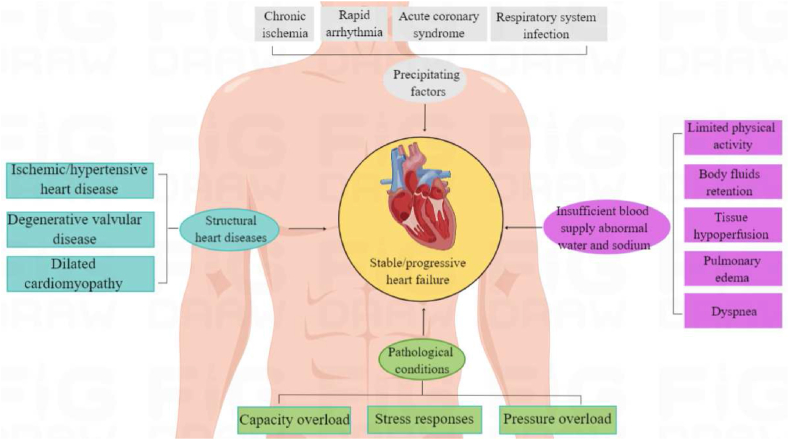
HF is a pathophysiological syndrome of various CVDs.

The cardiovascular disease (CVD) is the leading cause of death in the world, which is higher than tumor and other diseases with high morbidity, disability and mortality [[1], [2], [3]]. Coupled with the rapid development of social economy and the deepening of ageing, residents are generally exposed to the risk factors of CVDs, and the prevalence of CVDs is still on the rise [4,5]. In particular, heart failure (HF) is a pathophysiological syndrome including severe dysfunction of cardiac systolic and diastolic function, impaired ventricular filling and ejection function, and insufficient cardiac output caused by a variety of cardiac structural or functional diseases (Fig. 1) [[6], [7], [8]]. HF can produce a range of pathophysiological syndromes with clinical manifestations such as dyspnea, varying degrees of physical activity restriction and fluid retention, which is also the final stage in the development of various organic heart diseases, such as myocardial infarction, hypertension, aortic stenosis, valvular insufficiency and arrhythmia [9,10]. At present, there is no cure for HF, but only long-term medication can be employed to control the progression of HF. Therefore, summarizing the effective treatment of HF in the past is beneficial to understand the mechanism of action and develop new therapeutic drug [11,12].

There are complex physiological and pathological mechanisms in HF, and a variety of neurohumoral mechanisms including natriuretic peptide system, renin-angiotensin-aldosterone system (RAAS) and central nervous system (CNS) *etc* occupy the important position, while the inflammatory factors, endothelin and other humoral factors also contribute to the progression of disease [[13], [14], [15], [16], [17]]. However, the continuous in-depth researches on the pathogenesis of HF provide new progress in diagnosis and treatment. The treatment of HF is usually dominated by chemical drugs, which evolve from the traditional ‘cardiotonic, diuretic, vasodilator’ to the golden triangle combination therapy including the basis of aldosterone receptor antagonist (also known as mineralocorticoid receptor antagonist, MRA), angiotensin converting enzyme inhibitor (ACEI)/angiotensin II receptor blocker (ARB)/angiotensin receptor-neprilysin inhibitor (ARNI), and β receptor blocker [18,19]. The introduction of sodium-glucose cotransporter 2 inhibitor (SGLT-2i) constitutes of the new four couplets, which significantly reduce the risk of cardiovascular death or HF readmission [[20], [21], [22]]. In addition, on the basis of drug therapy, the mechanotherapy including cardiac resynchronization therapy (CRT) and implantable cardioverter defibrillator (ICD) *etc* further reduce the mortality and significantly improve the life quality and prognosis of patients [23,24]. However, the side effects and complications such as electrolyte disturbance, hypotension, bradycardia, poor prognosis and low life quality impose great burden on patients, which make the development of new treatments for HF highly challenging and desirable.

The natural products contain a variety of active ingredients such as alkaloid, glycoside, flavone and polyphenol *etc*, which exhibit diverse biological activities with the potential therapeutic value for HF [25,26]. Moreover, the natural products have the comprehensive advantages of high affinity, multi-target and multi-pathway that can avoid the single target induced various compensatory adverse reactions, as well as preventing the reduced pharmacological effects caused by the drug metabolism [27,28]. The representative natural products such as flavonoid, saponin, phenolic acid, alkaloid and polysaccharide have the effects of enhancing myocardial contractility, dilating blood vessels, diuresis and reducing myocardial remodeling, and the other biological activities including anti-fibrosis, anti-inflammation and anti-oxidative stress also contribute to HF therapy [29,30]. Compared with the reported chemical drugs, natural products and their active ingredients have several advantages: (1) provide new therapeutic targets and ideas for HF that could break through the current bottleneck of HF treatment; (2) offer the long-term efficacy with low toxicity and high safety; (3) prevent HF recurrence to improve the clinical symptoms and life quality [31,32]. Through comprehensively regulating the body function, immune function and self-adaptive ability of patients, natural products can reduce the body damage caused by various adverse reaction inducing factors, so as to reduce the rehospitalization or death of acute or chronic HF [33]. Since natural products have high affinity with multi-targets and exhibit potential activity in several mechanism of actions, the identification of exact mechanisms of related active ingredients is beneficial to develop drug candidates for the further clinical applications [34]. In this review, we will introduce the active natural products for the treatment of HF into several categories including flavonoid, saponin, phenolic acid, alkaloid and polysaccharide *etc* based on their structural scaffolds and mechanism of actions, and simply introduce the approved traditional Chinese medicine (TCM) against HF. We hope this timely and comprehensive review about natural product treatment for HF can provide an inspiration for the development of more effective anti-HF drugs with high safety and good drug-likeness.

## 2 Methodology and strategy

This review mainly focused on the structures, *in vitro* and *in vivo* evaluations of the natural products against HF. Firstly, we found the common names, active ingredient and the relevant plants based on the reference books and China National Knowledge Infrastructure (CNKI). Secondly, the references about natural products with anti-HF activity were collected throughout the PubMed, SCI-Finder, Web of Science, Google Scholar and CNKI using the relevant keywords including the names (Pueraria, epimedium and ginseng *etc*), active ingredient (flavonoid, saponin and phenolic acid *etc*), mechanism of actions (PI3K, MAPK and NF-κB *etc*). Finally, the most relevant research studies in the past five years were summarized and expected.

## 3 Classification of heart failure

The new guidelines essentially uphold the definition of previous guidelines: HF is a complex clinical syndrome characterized by manifestations and indications resulting from disorders in ventricular contraction or filling due to structural or functional abnormalities in heart. Different from the previous concept of ‘diastolic dysfunction’, greater emphasis was placed on the characterization of ‘filling disorder’. The new guidelines still categorize HF based on left ventricular ejection fraction (LVEF), where LVEF ≤40 % is referred to as HF with reduced ejection fraction (HFrEF), and LVEF ≥50 % is termed as HF with preserved ejection fraction (HFpEF). HF with borderline ejection fraction characterized by a LVEF between HFrEF and HFpEF can also derive eventual benefits from guide-directed drug therapy (GDMT), thus the new guidelines renaming this condition as HF with midrange ejection fraction (HFmrEF). The dynamic nature of LVEF necessitates continuous and dynamic observation and evaluation in HFmrEF, rather than a single measurement of EF value, to provide more meaningful insights. Moreover, due to the nonspecific nature of signs and symptoms associated with HF, which often overlap with other clinical manifestations, the diagnosis of HFmrEF and HFpEF should include the evidence of elevated left ventricular filling pressure in addition to typical clinical indicators.

### 3.1 Heart failure with reduced ejection fraction

HFrEF is one of the main types of chronic HF, with the long-term standardized drug therapy is the cornerstone of management. In this context, the three key therapeutic objectives for HFrEF encompass reducing mortality, preventing re-hospitalization due to worsening HF, and enhancing clinical status, functional capacity, and quality of life. Among the three categories of HF distinguished by LVEF, solely HFrEF currently possesses a pharmacological regimen that exhibits sufficient clinical evidence to enhance prognosis. In the management of HFrEF, ACEI/ARB and β receptor blocker are initially administered, followed by MRA, with each medication being titrated gradually from a low initial dose to reach the target dose or maximum tolerated dose. For patients who continue to experience symptoms despite treatment with conventional ‘Golden Triangle’ drugs, alternative medications such as ARNI and SGLT-2i may be considered.

### 3.2 Heart failure with preserved ejection fraction

In contrast to the well-established research on HFrEF, the management of HFpEF remains in an investigational stage. Numerous clinical trials have demonstrated that conventional therapeutic medications, such as ACEI/ARB, β receptor blocker, MRA and digoxin *etc* solely ameliorate symptoms without influencing the prognosis of HFpEF. In comparison to HFrEF, patients with HFpEF demonstrate a higher prevalence of comorbidities, including hypertension, atrial fibrillation, chronic kidney disease, type 2 diabetes, obesity and coronary heart disease. Therefore, it is imperative to prioritize screening for and effectively managing the underlying etiologies and comorbidities in order to optimize the clinical prognosis of patients with HFpEF. The pathophysiological mechanism of HFpEF is intricate, characterized by left ventricular diastolic dysfunction and ventricular remodeling resulting from inflammation and myocardial fibrosis. Consequently, interventions targeting the NO-soluble guanylate cyclase-cGMP (NO-sGC-cGMP) pathway have the potential to enhance myocardial and vascular function while decelerating ventricular remodeling.

### 3.3 Heart failure with midrange ejection fraction

Currently, there is limited attention devoted to HFmrEF, which encompasses approximately 10 %–20 % of patients diagnosed with HF. Traditionally, HFrEF and HFpEF have been attributed to systolic and diastolic dysfunction, respectively. The distribution of biomarkers in patients with HFrEF primarily correlates with cardiac remodeling, whereas in patients with HFpEF predominantly associates with inflammatory processes. However, emerging research suggests a growing recognition of substantial overlap between these two conditions. Compared to patients with HFpEF, those with HFmrEF are more prone to ischemic cardiomyopathy and hypertensive heart disease, resulting in a higher mortality rate compared to HFpEF patients with more reversible factors. However, the likelihood of ischemic cardiomyopathy in HFmrEF is similar to that in HFrEF, while HFrEF is more commonly associated with comorbidities such as hypertension and diabetes. Therefore, HFmrEF may share similarities in etiology with HFrEF while exhibiting a prognosis that aligns closer to that of HFpEF. The pathophysiological mechanism of HFmrEF is distinct, and its etiology, clinical characteristics, treatment, and prognosis differ from those observed in the other two types of HF. Consequently, HFmrEF represents a promising avenue for future research in the field of HF.

## 4 Pathologic mechanisms of heart failure

**Fig. 2:**
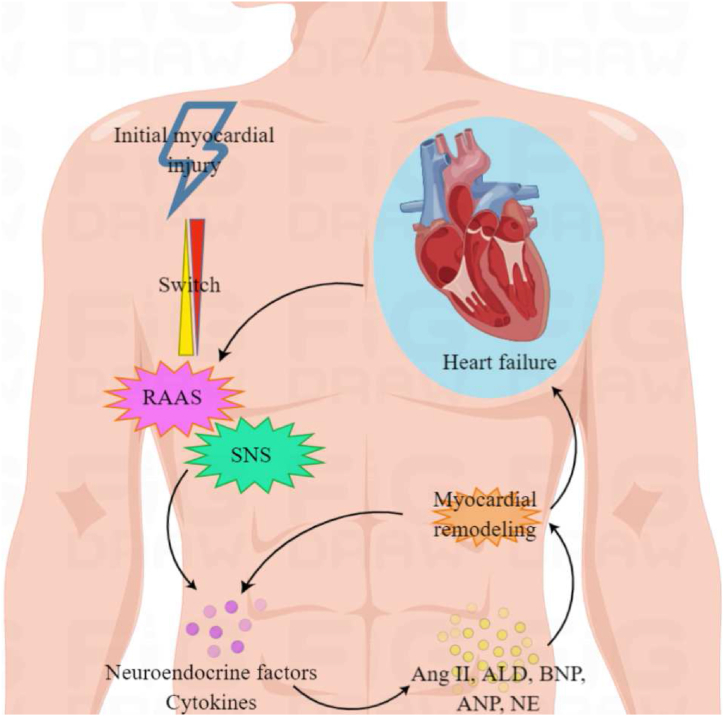
The pathologic mechanism of HF.

At the initial stage of myocardial injury, the sympathetic nervous system (SNS), RAAS and natriuretic peptide system (NPS) *etc* are activated to release a variety of related endogenous neuroendocrine factors and cytokines [35]. The long-term and chronic activation of these factors promotes myocardial remodeling, thus exacerbating myocardial damage and worsening cardiac function, which in turn further activates neuroendocrine factors and cytokines to form a vicious cycle (Fig. 2) [36]. For example, the activation of angiotensin II (Ang-II) can promote the norepinephrine (NE) release and increase the excitability of α1 receptor, lead to the myocardial fibrosis, hypertrophy, and deformation through activating the proto-oncogene expression, increasing the synthesis of proteins and microRNAs and regulating the function of mRNAs in cardiomyocytes [37]. In particular, the elevated NE concentration can stimulate β receptor and α1 receptor, which further promote the myocardial hypertrophy formation and cardiac deterioration through regulation of RASS system [38]. And the overexposed β receptor will cause the abnormal signal transduction to affect the systole and diastole through damaging the Ca^2+^ uptake and release of cardiomyocyte. In addition, other mechanisms and associated biomarkers such as interleukin (IL), tumor necrosis factor α (TNF-α), brain natriuretic peptide (BNP), transforming growth factor β (TGF-β) and heart fatty acid binding protein (HFABP) *etc* also contribute to HF formation [39,40]. Therefore, the mechanism of HF is the neurohumoral regulation and myocardial remodeling induced synergistic result of various pathogenetic interactions, such as the activation of RAAS system, excitation of SNS, apoptosis, autophagy, inflammatory cytokines and oxidative stress *etc.*

### 4.1 Ionic defect

The most basic systole is regulated by ion exchange, in which Na^+^/Ca^2+^ exchange is the most critical. The increased Ca^2+^ level can improve HF to some extent, but enhance the risk of arrhythmia, myocardial hypertrophy and myocardial apoptosis, which indicates that Ca^2+^ is crucial for HF. The ionic defect leads to the reduced systolic function and activates the abnormal expression of potassium channels, which can further aggravate myocardial remodeling through various mechanisms and result in HF [41]. In addition, the recombination of sarcoplasmic reticulum is involved in the occurrence of HF, and the alteration of endoplasmic reticulum structure suggests abnormal Ca^2+^ conversion.

### 4.2 Inflammation

The inflammatory response is one of the key steps in myocardial repair [42]. In particular, leukocytes participate in the clearance of necrotic cardiomyocytes and initiate the process of myocardial injury repair. The ischemic cardiomyocytes release adenosine triphosphate (ATP), which chemotaxis macrophages to remove the dead cells and debris in the inflammatory areas. The ATP released by central granulocytes will chemotaxis other central granulocytes to the inflammatory areas, and eventually promote myocardial repair through regulating immune cells and ischemia-reperfusion (I/R) injury [43]. The immune activation and inflammatory responses play important roles in the progression of HF, which are marked by the increased inflammatory cells. In particular, the IL-1β can reduce myocardial contractility and reshape myocardium, while the levels of TNF-α and IL-10 are significantly elevated in HF patients. The chemokines released by myocardial injury and apoptotic cells may cause the aggregation of inflammatory cytokines in necrotic and non-necrotic areas, lead to the occurrence of myocardial damage and cardiac insufficiency through promoting the fibroblast activation and extracellular matrix degradation and improving the activity of matrix metalloproteinase (MMP) [44]. After myocardial infarction, macrophages become the main source of MMP-28 synthesis and secretion, and the related modifications lead to myocardial remodeling and cardiac function decreasing, thereby increasing the risk of adverse events. Moreover, the dysfunction of Th1/Th2 cells may be one of the mechanisms of HF caused autoimmune stress, and participates in the process of HF-induced myocardial injury.

### 4.3 Neurohumoral regulation

In the progress of HF, the SNS and RAAS are activated to increase the myocardial contractility and cardiac output, while constricting peripheral blood vessels to maintain normal arterial blood pressure and blood supply for vital organs [45]. Although such changes can temporarily regulate and maintain the circulation, the long-term overload will damage the cardiovascular system via causing myocardial hypertrophy. Moreover, the overload activation of RAAS in patients with HF can promote vascular and myocardial remodeling, further aggravating myocardial injury and worsening cardiac function [46].

### 4.4 Apoptosis

The continuous loss of cardiomyocytes is the main cause of progressive loss of left ventricular function in the program of HF, in which apoptosis is the main form of cell loss [47]. Cardiomyocyte apoptosis is involved in the development and progression of HF, and it may signal the decompensation of cardiac function. There are many factors that stimulate cells to apoptosis, such as myocardial hypoxia, reperfusion injury, ischemia and pressure volume overload *etc* [48]. Therefore, several reported natural products could inhibit cardiomyocyte apoptosis and play a role in myocardial protection through regulating the expression levels of apoptotic proteins including B-cell lymphoma-2 (Bcl-2), Bcl-2-associated X protein (Bax), cysteine aspartate-specific protease-3 (caspase-3) and nuclear factor kappa-B (NF-κB) *etc* [49].

### 4.5 Autophagy

The moderate autophagy may participate in the cleaning of excess substances in cells and exert a stable myocardial protective effect. However, the excessive autophagy may activate the cytokine system leading to cardiac cell death and further aggravate the development of HF [50]. The autophagy in heart mainly depends on the lysosome pathway, including the phagophore formation, autophagosome formation, autophagolysosome formation and degradation. There are two main pathways for autophagy generation, which are autophagy dependent on mammalian target of rapamycin (mTOR) and autophagy independent of mTOR. In particular, the mTOR dependent autophagy can be divided into the following three categories: (1) phosphoinositide 3-kinase (PI3K)/protein kinase B (Akt)/mTOR signaling pathway; (2) adenosine 5′-monophosphate (AMP)-activated protein kinase (AMPK) signaling pathway; (3) mitogen-activated protein kinase (MAPK) signaling pathway. On the other hand, the autophagy pathways independent of mTOR include different types of PI3K pathway, Beclin-1 pathway and p53 signaling pathway *etc*. In addition, miR-221 is an important regulator of autophagy balance and myocardial remodeling, which may become a new target for the treatment of HF [51]. Mitochondrial autophagy is a form of selective autophagy that eliminates the dysfunctional mitochondria within cells by autophagy, which can be categorized into ubiquitin-dependent and non-ubiquitin-dependent pathways. Mitochondrial autophagy plays a crucial role in maintaining cardiomyocyte homeostasis by timely eliminating damaged mitochondria and preventing the toxic impact of reactive oxygen species (ROS) on cellular function. However, the sustained upregulation of mitochondrial autophagy may lead to excessive clearance of mitochondria, resulting in HF due to a reduction in muscle cell mitochondria and an inability to generate sufficient ATP for maintaining the continuous contractile function of heart. Currently, the signaling pathways associated with mitochondrial autophagy involve phosphatase and tensin homolog (PTEN)-induced kinase 1 (PINK1)/Parkin, Bcl-2 interacting protein 3 (BNIP3)/Nip3-like protein X (Nix), FUN14 domain-containing protein 1 (FUNDC1), PI3K/Akt, as well as end-sequence proteins such as Atg32 *etc*. In particular, the PINK1/Parkin signaling pathway, extensively investigated in the context of mitochondrial autophagy, plays a pivotal role in regulating mitochondrial function, facilitating mitochondrial autophagy, and preserving cardiomyocyte morphology. In the pathological state, mitochondrial depolarization impedes the translocation of PINK1 into mitochondria, leading to its activation on the outer mitochondrial membrane through self-phosphorylation. Subsequently, the activated PINK1 recruits and acidifies Parkin, thereby activating its E3 ligase activity and facilitating ubiquitination of various mitochondrial surface proteins including mitofusin 1/2 (Mfn1/2) and voltage-dependent anion channel-1 (VDAC-1), ultimately promoting mitophagy. In conclusion, excessive activation or excessive inhibition of autophagy may lead to further exacerbation of HF. Therefore, how to maintain the balance and stability of autophagy is a major focus to ensure the stability of myocardium.

### 4.6 Mitochondrial homeostasis

The mitochondria within the cell are interconnected, forming a three-dimensional tubular network structure comprising an outer membrane, a highly folded inner membrane that creates ridges, and a membrane space between the bilayer membranes. The mitochondria undergo frequent fusion and division, exhibiting both morphological and kinetic characteristics that are subject to constant adjustment and modification in response to changes in the cell cycle as well as internal and external environments. Mitochondrial dynamics play crucial roles in various biological processes, encompassing ATP synthesis, mitochondrial DNA (mtDNA) inheritance, Ca^2+^ signaling transduction, cellular division, autophagy, apoptosis and senescence. The regulation of mitochondrial dynamics primarily relies on five key protein molecules, namely Mfn1, Mfn2, optic atrophy-1 (OPA1), dynamin-related protein 1 (Drp1) and mitochondrial fission 1 protein (Fis1). Among these proteins, Mfn1, Mfn2 and OPA1 play crucial roles in governing the process of mitochondrial fusion, while Drp1 and Fis1 are responsible for mediating the process of mitochondrial division. Additionally, TNF-α receptor 2, the mitochondrial permeability transition pore (mPTP), and the mitochondrial calcium uniporter (MCU) actively contribute to maintaining optimal mitochondrial homeostasis and membrane potential. Consequently, they enhance both mitochondrial morphology and function while effectively impeding the progression of HF. The inadequate energy supply or metabolic disorders of myocardium often damage the structure and function of the mitochondria and force the myocardium to overwork, resulting in the cardiac injury [52]. The AMPK/peroxisome proliferators-activated receptor α (PPAR-α) plays an important role in promoting mitochondrial biogenesis and improving energy metabolism of failing myocardium. In particular, AMPK is the valve of biological energy metabolism and is related to mitochondrial biogenesis. PPAR is a ligand-activated receptor in nuclear hormone receptor family, among which, PPAR-α is the downstream target of this signaling pathway and is positively correlated with the expression of AMPK [53]. It is mainly involved in the oxidation of fatty acids, promotes the expression of various fatty acid metabolic enzymes and genes, and improves the utilization of fatty acids. The AMPK/PPAR-α signaling pathway can promote the expression of glucose transporter type 4 (GLUT-4), regulate glycogen synthase kinase-3β (GSK-3β) to inhibit glycogen synthesis, and ensure the supply of substrates for myocardial energy metabolism [54]. Moreover, the abnormal blood oxygen supply and demand, mitochondrial damage and abnormal mitochondrial oxidative phosphorylation function will further aggravate HF.

### 4.7 Epigenetic inheritance

Myocardial hypertrophy is a compensatory expression of myocardial injury, which can increase cardiac work and improve myocardial systolic function. However, long-term myocardial hypertrophy can lead to interstitial fibrosis, cardiomyocyte apoptosis, and eventually HF [55]. In the normal conditions, the histone deacetylases (HDACs) can inhibit the expression of myocardial hypertrophy genes. However, the related factors (protein kinase D and G protein coupled receptor kinase 5 *etc*) will be stimulated during myocardial hypertrophy, thus activating HDACs and causing translocation of HDACs to interact with the related transcription factors and weaken the inhibitory effect against myocardial hypertrophy [56]. Therefore, limitation of the stimulators that activate HDACs may be beneficial in preventing the development of HF through regulation of metabolic disorders and myocardial hypertrophy. In addition, the myofibroblast-specific lysine-specific demethylase 1 (LSD1) deletion attenuates transverse aortic constriction-induced myocardial remodeling and pressure overload-induced myocardial fibrosis to delay the progression of HF and improve cardiac function [57].

### 4.8 Vascular dysfunction

The pathogenesis of HFpEF may be due to the coronary microvascular dysfunction, which also leads to an increased oxidative stress response that restricts NO-cGMP-PKG [58]. The NO-cGMP-PKG signaling contributes to overall cardiomyocyte hypertrophy and dysfunction in two main signaling pathways including NO-soluble guanylate cyclase-cGMP (NO-sGC-cGMP) and natriuretic peptide-particulate guanylate cyclase-cGMP (NP-pGC-cGMP) signaling pathways. The increasement of left ventricular filling pressure (LVFP) caused by abnormal diastolic function is also a main reason leading to HFpEF, which could be divided into hemodynamic mechanism (diastolic dysfunction, left ventricular congestion and pulmonary vascular disease *etc*) and potential molecular mechanism (microvascular inflammation, abnormal cardiac metabolic function and abnormal cell structure caused by connection or fibrosis) [59].

### 4.9 Oxidative stress

The oxidative stress phenomenon is characterized by an excessive generation of ROS and a concomitant reduction in antioxidant capacity, resulting in oxidative damage. Notably, this phenomenon is significantly amplified in the context of HF, where an upregulation of ROS production has been observed under conditions of ischemia or hypoxia. ROS can induce damage to macromolecules within mitochondria or impede their synthesis, while mitochondria, being a crucial source of ROS, are also susceptible to ROS-induced impairment. The absence of robust chromatin structure protection, limited capacity of mtDNA for repairing DNA damage, and impermeability of the mitochondrial membrane to superoxide anions collectively contribute to extensive organelle damage induced by ROS. The elevated levels of ROS in failing cardiac tissues are associated with mitochondrial impairment and dysfunction, primarily characterized by heightened mitochondrial lipid peroxidation, reduced mtDNA copy number, diminished mtRNA transcription, impaired respiratory chain complex enzyme activity, and compromised oxidative capacity. These alterations collectively contribute to a detrimental cycle of declining mitochondrial function, increased ROS production and exacerbated cellular damage. Moreover, oxidative stress plays an increasingly pivotal role in cardiac remodeling, with myocardial remodeling serving as the primary mediator of HF progression. The activation of tyrosine kinase Src, guanosine triphosphate (GTP) binding protein Ras, protein kinase C (PKC), MAPK, and c-Jun N-terminal kinase (JNK) in the ROS signaling pathway leads to the induction of apoptosis in cardiomyocytes. Through mediating DNA and mitochondrial damage and activating apoptotic signaling pathways, ROS induces cardiomyocyte apoptosis, myocardial remodeling, and dysfunction. Moreover, through activation of poly(ADP-ribose)polymerase-1 (PARP-1) to modulate the expression of diverse inflammatory factors, ROS can induce DNA damage and expedite myocardial remodeling.

## 5 The development of drug therapy for heart failure

Over the past five decades, the paradigm of drug therapy for HF has evolved significantly from correcting hemodynamics to blocking neuroendocrine overactivation and further multi-mechanism/target therapy with the deeper understanding of HF. Early, the conventional treatment of HF mainly focused on cardiotonic, diuretic and vasodilator, which could be used to enhance myocardial contractility, to improve edema, to reduce pre and post cardiac load, respectively [60]. These drugs have good short-term effects, but can’t prevent the progression of HF or significantly reduce mortality. Subsequently, numerous studies have confirmed that myocardial remodeling caused by activation of the neuroendocrine system is a key factor causing the occurrence and development of HF. Therefore, the golden triangle combination of ACEI/ARB, β receptor blocker and MRA have been used to treat HF through inhibiting the overexcitation of RAAS and the sympathetic nervous system [61]. Recently, the treatment of HF has entered a multi-mechanism and multi-target stage with the introduction of new drugs, including ARNI (instead of ACEI/ARB in golden triangle combination), SGLT-2i, sGC agonist, the cardiac hyperpolarization-activated funny current (I_f_) inhibitor and myosin agonist *etc* [62]. The emergence of new drugs has opened up a new situation in the treatment of HF.

### 5.1 Cardiac glycoside

Cardiac glycosides, a class of organic natural products, are commonly used in the treatment of HF and arrhythmia. Exogenous cardiac glycosides for the treatment of HF mainly come from the family of *Scrophulariaceae* and *Nerium oleander*, such as lily of the valley, oleander and digitalis *etc* [63]. At present, the clinical use of cardiac glycosides includes digitoxin, digoxin, lanatoside and strophanthin K *etc*, which are composed of sugar residue, unsaturated lactone ring and steroidal nuclei. In particular, digitalis is primarily employed to treat HF with reduced ejection fraction (HFrEF) in patients after the use of diuretics, ACEI/ARB, β receptor blocker and MRA [64]. The pharmacological effect of cardiac glycosides on HF is based on increasing Ca^2+^ concentration in cardiomyocytes, and the relevant mechanisms can be divided into three aspects: (1) the specific binding and inhibition of Na^+^-K^+^-ATPase (NKA); (2) the change of sodium-calcium exchanger (NCX) drive; (3) the subsequent ryanodine receptor/sarcoplasmic reticulum (RyR/SR)-related calcium store release mechanism [65].

### 5.2 Diuretic

The excess circulating capacity caused by increased extracellular fluid is an important pathogenesis of HF. Therefore, the early removal of excess body fluid is the main therapeutic strategy to alleviate clinical symptoms, in which diuretics including loop diuretic, thiazide diuretic and MRA can effectively reduce the volume load by promoting the excretion of water and sodium in kidney [66]. Clinically, there are many kinds of diuretics, which can be divided into three categories according to the site of action: (1) interfere with the K^+^-Na^+^-2Cl^-^ transport system and play a strong diuretic role; (2) affect the Na^+^-Cl^-^ transport system at the proximal end of distal convoluted tubule with the moderate intensity; (3) block the Na^+^ channel at collecting tubule and the end of collecting tubule [67]. However, the efficacy of therapeutic diuretics is challenged by the diuretic resistance, which does not effectively reduce edema despite the use of appropriate or increasing the dosage of diuretics. The mechanism of diuretic resistance is complex, which may be related to the side effects of diuretics such as electrolyte disturbance, glucose metabolism and uric acid metabolism as well as the renal insufficiency, renal tubular cell hypertrophy and drug interaction *etc*. There are many clinical methods for the treatment of diuretic resistance, such as changing the drug usage to intravenous medication or increasing the drug dose, which can also be solved by combination drug therapy. The novel diuretics like the selective arginine vasopressin (AVP) receptor 2 antagonist tolvaptan can block the binding of AVP to V2 receptor (V2R) of renal tubule cells to increase the free water excretion and serum sodium concentration [68]. In addition, the specific adenosine A1 receptor (ADORA1) antagonist KW3902 can reduce the pressure of renal afferent arterioles and inhibit the reabsorption of sodium by proximal convoluted tubules to increase the excretion of water and sodium and delay the deterioration of renal function.

### 5.3 Vasodilator

Vasodilators can dilate the peripheral blood vessels by directly dilating vascular smooth muscle (VSM) or acting on adrenergic receptors, which can lower blood pressure, reduce peripheral vascular resistance and cardiac load and stabilize hemodynamics to improve the clinical symptoms of HF [69]. The sGC agonists such as vericiguat, cinaciguat and praliciguat can bind to the non-heme site, activate sGC independently of NO and catalyze the conversion of guanosine triphosphate into cGMP, lead to the effects of enhancing vasodilatation, lowering blood pressure, inhibiting platelet aggregation, tissue fibrosis and smooth muscle proliferation. The nitric ester drugs like isosorbide mononitrate can increase the release of NO in patients with acute HF to promote the increase of intracellular guanylic acid, as well as inhibiting Ca^2+^ influx to relax VSM [70]. The BNPs including nesiritide, uralitide and capericide *etc* can activate sGC by binding to the sGC receptor on the cell membrane to increase the content of cGMP in the cell and produce the biological effects (like diuresis, sodium excretion and vasodilation) of various natriuretic peptides. The relaxin-2 can reduce the systemic vascular resistance, increase the arterial compliance, cardiac output and renal blood flow, and increase the concentration of NO through regulating the G protein-coupled receptor (GPCR)-related signaling pathways to prevent fibrosis [71]. Sodium nitroprusside can improve the ventricular load and kidney blood flow, which plays a direct role in the arteries and veins to expand VSM and reduce vascular resistance.

### 5.4 Angiotensin converting enzyme inhibitor (ACEI)

The real cause of HF is neuroendocrine-mediated myocardial remodeling, which is characterized by cardiomyocyte hypertrophy, cardiomyocyte apoptosis and changes in the extracellular cardiac matrix (ECM) [72]. ACEIs such as captopril, enalapril and benazepril can block the process of myocardial remodeling in patients with HF by inhibiting the activation of neuroendocrine system (especially the formation of Ang-II and aldosterone) to inhibit the sympathetic nerve activity and improve the parasympathetic nerve activity. The mechanism of action of ACEI is to inhibit the production of Ang-II and reduce the degradation of vascular bradykinin. In particular, Ang-II is a powerful vasoconstrictive factor that damage to the failing heart by increasing oxygen consumption, promoting cell growth, stimulating vascular and left ventricular remodeling, and producing proteinuria [73]. Meanwhile, the vascular bradykinin can stimulate the release of NO and prostacyclin. These two potent vasodilator factors can be used to explain the beneficial mechanism of ACEI in hemodynamics.

### 5.5 Angiotensin II receptor blocker (ARB)

ARBs can inhibit the sympathetic nerve activity and renin activity and reverse ventricular remodeling like ACEIs, but their specific drug effects are not exactly the same as ACEI. Although ACEI plays a role in the treatment of HF by regulating the function of bradykinin, vascular bradykinin is also responsible for the adverse effects of ACEI such as cough, kidney dysfunction and angioneurotic edema [74]. ARBs including losartan, valsartan and irbesartan *etc* can inhibit the binding of Ang-II receptor through ACE-dependent and independent pathways, thereby inhibiting RAAS without increasing the blood concentration of bradykinin [75]. The incidence of ARBs adverse reactions such as cough and angioneurotic edema is significantly lower than that of ACEIs, so they are mainly used in cardiovascular patients who can’t tolerate ACEIs [76].

### 5.6 β receptor blocker

β Receptor blockers are widely used in the treatment of HF, myocardial infarction, hypertension and other diseases, which can selectively bind β-adrenergic receptor to play an antagonistic role of neurotransmitters and catecholamines [77]. The representative β receptor blockers like bisoprolol and metoprolol *etc* work in the treatment of HF by the following mechanisms: (1) block the myocardial toxicity of catecholamines; (2) increase the number of β1 receptor and restore the myocardial sensitivity to catecholamines to improve the cardiac function; (3) slow down heart rate, reduce myocardial oxygen consumption and increase myocardial energy reserve to improve the myocardial diastolic function, reverse pathological ventricular remodeling and increase cardiac output; (4) play an anti-arrhythmic role and reduce the rate of sudden death; (5) inhibit the overactivation of RAAS to dilate blood vessel, reduce water and sodium retention and reduce cardiac load [78]. Moreover, according to the different selectivity of β receptor blockers, they are mainly divided into three classes: (1) the non-selective β receptor blockers that block β1 and β2 receptors and increase the vascular resistance in peripheral arteries (propranolol); (2) the selective β1 receptor blockers that only block β1 receptor and have little effect on β2 receptor (metoprolol and bisoprolol); (3) the selective β receptor blockers with peripheral vasodilation function that act on both β and α1 receptors to dilate the peripheral blood vessels (carvedilol) [79]. In general, β receptor blockers shouldn’t be used until HF is stable, and the patients should be referred to whether anti-HF drugs such as intravenous cardiotonic, diuretic and vasodilators *etc* are used.

### 5.7 Mineralocorticoid receptor antagonist (MRA)

MRAs not only have significant effects on HF and refractory hypertension, but also on acute myocardial infarction, kidney disease and metabolic syndrome [80]. The representative MRAs like spironolactone, eplerenone and finerenone *etc* can block the overactivation of neuroendocrine system in all stages of HF and inhibit the occurrence and development of myocardial remodeling. MRAs can significantly inhibit collagen synthesis and alleviate myocardial fibrosis, which is significantly better than that of angiotensin receptor antagonists ACEIs and ARBs [81]. In addition, MRAs can regulate the balance of K^+^ and Mg^2+^ in myocardium and improve the diastolic and systolic functions to improve myocardial remodeling without affecting the repair of myocardial tissue and scar formation [82]. Therefore, MRAs can help ACEIs and ARBs to control the level of aldosterone, reduce myocardial and vascular fibrosis, improve ventricular remodeling and reduce the mortality and morbidity of patients with HF to become an important medicine for the treatment of HF.

### 5.8 Angiotensin receptor-neprilysin inhibitor (ARNI)

The emergence of a new generation of anti-HF drugs ARNI has brought a new direction for the treatment of HF [83]. ARNI like sacubitril/valsartan can inhibit myocardial remodeling by inhibiting RAAS and neprilysin, inhibit the expression of chromogranin A (CgA) to inhibit ventricular remodeling, and reduce myocardial fibrosis by inhibiting TGF-β expression [84]. Moreover, ARNIs can be used to treat patients who are resistant to ACEIs or ARBs and is expected to become a cornerstone drug in the treatment of chronic HF [85].

### 5.9 Sodium-glucose cotransporter 2 inhibitor (SGLT-2i)

The glucoside ligand of new hypoglycemic drug SGLT-2i can competitively binds to the glucose-binding end of the transporter and reduce blood glucose by blocking glucose reabsorption in the proximal renal tubules [86]. In addition to the hypoglycemic effects, SGLT-2i also has the significant effects on cardiovascular protection [87]. In particular, the treatment of HF with representative SGLT-2i including empagliflozin, canagliflozin and dapagliflozin *etc* does not depend on the hypoglycemic effect, and mainly works through the following mechanisms: (1) promote osmotic diuresis and reduce water and sodium retention; (2) inhibit sympathetic nerve activity and promote the body to return to hemodynamic homeostasis; (3) change myocardial energy metabolism, increase serum erythropoietin (EPO) concentration, enhance ketone body content and availability; (4) delay or reverse ventricular remodeling, resist inflammatory response and reduce myocardial fibrosis; (5) play a comprehensive role in regulating metabolism, reduce weight, blood pressure and uric acid [88,89]. From a single hypoglycemic to a comprehensive regulation of metabolic homeostasis, SGLT-2i is expected to become a potential drug for the co-treatment of cardiovascular and metabolic diseases.

### 5.10 Other drugs

In addition to the above anti-HF drugs, some novel drugs have been developed for the treatment of HF and related complications. For example, the selective I_f_ inhibitor ivabradine can inhibit the diastolic depolarization of sinoatrial node, thus reducing the frequency of sinoatrial node without affecting the blood pressure, myocardial contractility and conductivity [90]. The calcium ion sensitizer levosimendan can bind with cardiac troponin C to produce positive inotropic effects, open ATP-sensitive K^+^ channel to produce vasodilation effect, and inhibit phosphodiesterase III to enhance myocardial contractility and reduce anterior and posterior load without affecting diastolic function [91]. The selective myocardial myosin agonist omecamtiv mecarbil (OM) can activate the myocardial myosin S1 subunit, increase ATP turnover rate and accelerate actin-myosin circulation to improve the myocardial contractility [92]. Moreover, other drugs like the glucagon-like peptide-1 (GLP-1) receptor agonist, chalybeate, calcium channel blocker and coenzyme Q10 *etc* also have the anti-HF function, which can lead to further breakthroughs in the treatment of HF [93,94].

## 6 The representative natural products with anti-heart failure activity

Natural medicines contain a variety of chemical components, such as alkaloids, polysaccharides, glycosides, flavonoids and enzymes *etc*, which may be the active compounds with therapeutic value [95]. Natural products have the characteristics of multi-pathway and multi-target comprehensive efficacy, which can avoid multiple compensatory adverse reactions caused by a single target and the weakening of pharmacological effects caused by drug metabolism (Table 1). In particular, natural products can improve the clinical symptoms and life quality, provide new therapeutic targets and ideas for anti-HF, regulate the cardiac function and prevent the recurrence of HF with high efficacy and low toxicity to breakthrough the current bottleneck of HF therapy [96]. Moreover, the early adjuvant treatment of natural products can reduce the use of digitalis, diuretics and other anti-HF drugs, and reduce the adverse reactions caused by the long-term use of drugs. For example, many natural plants including *Aconitum carmichaeli Debx*, *Rhizoma Chuanxiong* and *Astragalus membranaceus* have the effects of enhancing myocardial contractility, dilating blood vessels, diuresis and reducing ventricular remodeling.

**Table 1:** The representative natural products for the treatment of heart failure.

Natural product	Active ingredient	Source	Target and related mechanism	Effect	Ref.
Flavonoid	Puerarin	*Pueraria montana*	Channel current I_Na_, I_Ca-L_ and K_ATP_; Nrf2, Keap1, heme oxygenase and glutathione S-transferase; NF-κB, ERK1/2 and p38MAPK pathways.	Myocardial ischemia and arrhythmia; Myocardial fibrosis; Myocardial hypertrophy.	[100,101]
	Icariin	*Epimedium brevicornum*	MMP 2/9 and collagen; GRP78, GRP94, CHOP, caspase-3 and ROS; TGF-β1/Smad2 pathway, α-SMA and MMPs/MMP-1.	Ventricular remodeling; Apoptosis; Myocardial fibrosis.	[[103], [104], [105]]
	Luteolin	*Reseda odorata*	TLR4/MyD88/NF-κB pathway, IL-6, IL-1β and TNF-α; SERCA2α, SOD, GSH-Px; Akt/STAT3 pathway; mTOR pathway.	Inflammation; Oxidative stress; Apoptosis; Autophagy.	[[107], [108], [109], [110]]
	Baicalin	*Scutellaria baicalensis*	Cyt C, Apaf-1, caspase-3/9 and CHOP/eNOS/NO pathway; PI3K/Akt/eNOS pathway, RIP1/3 and p-MLKL; PPAR β/δ.	Apoptosis; Energy metabolism; Myocardial hypertrophy.	[[112], [113], [114], [115]]
	Methylophi-opogonanone A	*Ophiopogon japonicus*	PI3K/Akt/eNOS pathway; SOD and catalase.	Myocardial ischemia; Apoptosis.	[117,118]
Saponin	Ginsenoside Re	*Panax ginseng*	Nrf2, GCLC, GCLM; LDH, CK and myocardial hydroxyproline; TGF-β/Smad3 pathway.	Apoptosis; Energy metabolism; Myocardial fibrosis.	[[122], [123], [124], [125], [126], [127]]
	Ginsenoside Rh1	*Panax ginseng*	NF-κB, TNF-α, IL-6 and ERK1/2 pathway; PI3K/Akt/MAPK pathway, MMPs	Inflammation; Apoptosis.	[[122], [123], [124], [125], [126], [127]]
	Ginsenoside Rb1	*Panax ginseng*	Caspase-3/9 and Bax protein; PINK1 and LC3-I/II; TGF-α/β, Ang-II and MAPK pathway; AMPK pathway.	Apoptosis; Autophagy; Myocardial hypertrophy; Energy metabolism.	[[122], [123], [124], [125], [126], [127]]
	Ginsenoside Rg1	*Panax ginseng*	HIF-1 and Sestrin1; TNF-β/JNK/MAPK pathway; TLR4/TRIF/IRF3 pathway.	Oxidative stress; Myocardial fibrosis; Inflammation.	[[122], [123], [124], [125], [126], [127]]
	Ginsenoside Rg2	*Panax ginseng*	SOD and MDA; Akt/mTOR pathway.	Myocardial infarction; Autophagy.	[[122], [123], [124], [125], [126], [127]]
	Ginsenoside Rg3	*Panax ginseng*	AMPK pathway; RAAS and Ang-II; TLR4/NF-κB/IL-1β pathway; TGF-β/Smad/NF-κB pathway.	Autophagy; Myocardial fibrosis; Inflammation; Apoptosis.	[[122], [123], [124], [125], [126], [127]]
	Ophiopogonin D	*Ophiopogon japonicus*	PI3K/GSK-3β pathway, caspase-3 and Bax proteins; PINK1/Parkin pathway; TNF-α and IL-1β.	Apoptosis; Mitochondrial function; Inflammation.	[[129], [130], [131]]
	Astragaloside	*Astragalus membranaceus*	PKC-α, SERCA2α and PPAR-α; NLRP3, IL-6 and TLR4.	Mitochondrial function; Myocardial fibrosis.	[[133], [134], [135], [136]]
	Momordicoside G	*Momordica charantia*	ANP, β-MHC and α-SKA; iNOS and COX-2.	Myocardial hypertrophy; Energy metabolism.	[[138], [139], [140]]
Phenol and phenolic acid	Salvianolic acid A	*Salvia miltiorrhiza*	LDH, JNK, ERK1/2 pathway; PGC-1α; BNP, MDA and MMPs.	Apoptosis; Mitochondrial function; Myocardial fibrosis.	[[144], [145], [146]]
	Salvianolic acid B	*Salvia miltiorrhiza*	PARP-1; ERK1/2 pathway, GATA4 and BNP	Mitochondrial function; Myocardial remodeling.	[147,148]
	Ferulic acid	*Ligusticum chuanxiong*	PKC/MAPK pathway, ERK1/2, Ang-II and endothelin-1; LDH, creatine kinase and cardia troponin; PI3K/Akt/mTOR pathway.	Myocardial hypertrophy; Myocardial infarction; Autophagy.	[[150], [151], [152]]
	Curcumin	*Curcuma longa*	SIRT1; DKK3, ASK1 and JNK.	Myocardial infarction; Apoptosis.	[154,155]
	Hydroxysafflor yellow A	*Carthamus tinctorius*	VEGF-A, MMP-9 and nucleolin; LDH; Caspase-3; PI3K/Akt pathway.	Myocardial ischemia; Oxidative stress; Apoptosis; Mitochondrial function.	[157,158]
	Chlorogenic acid	*Eucommiaceae* and *Caprifoliaceae*	TGF-β1/Smads pathway; Caspase-3, Bax, PI3K/Akt pathway.	Myocardial fibrosis; Apoptosis.	[[159], [160], [161]]
Alkaloid	Tetramethylpyrazine	*Ligusticum chuanxiong*	microRNA-499a, Sirtuin1; PI3K/Akt pathway.	Oxidative stress; Apoptosis.	[165,166]
	Liguzinediol	*Ligusticum chuanxiong*	Bcl-2, Bax, caspase-3 and NF-κB.	Apoptosis.	[168]
	Berberine	*Coptis chinensis*	AMPK and PI3K/Akt/NOS pathways; MAPK and Akt pathways; Notch1/Hes1-PTEN/Akt pathway.	Apoptosis; Autophagy; Myocardial injury.	[[170], [171], [172]]
	Higenamine	*Aconitum carmichaeli*	LKB1/AMPK/SIRT1 pathway.	Mitochondrial function.	[174]
	Aconitine	*Aconitum carmichaeli*	PPAR-α/PGC-1α/SIRT3 pathway; RAAS and other neuropoietic cytokines.	Energy metabolism; Ventricular remodeling.	[[176], [177], [178]]
	Morphine	*Papaver somniferum*	PKCα, ERK and MAPK pathways.	Myocardial infarction.	[180,181]
Polysaccharide	Astragalus polysaccharide	*Stragalus membranaceus*	TNF-β/PGC-1α pathway, NFATC3 and CaMK-II; PPAR-γ and TGF-β1.	Myocardial hypertrophy; Myocardial fibrosis.	[[185], [186], [187]]
	Pachymaran	*Poria cocos*	BNP, AVP/V2R/AQP-2 pathway.	Myocardial protection.	[[189], [190], [191]]
	Ophiopogon japonicus polysaccharide	*Ophiopogon japonicus*	SOD, GSH-Px and MDA.	Oxidative stress.	[193,194]
	Lycium barbarum polysaccharide	*Lycium chinense*	LDH and Bax; microRNA-1.	Apoptosis; Myocardial remodeling.	[196,197]
Others	Cycloastragenol	*Astragalus membranaceus*	BNP and Ang-II; MMP2/9; Akt/RPS6KB1 pathway.	Myocardial injury; Myocardial remodeling; Autophagy.	[200]
	Pachymic acid	*Poria cocos*	TNF-α, IL-1 and IL-6; Caspase-3/6/9.	Inflammation; Apoptosis.	[[202], [203], [204]]
	Tanshinone IIA	*Salvia miltiorrhiza*	MAP1LC3, SQSTM1, LAMP-1 and Beclin-1; AMPK/mTOR pathway.	Autophagy; Apoptosis.	[[206], [207], [208]]
	Schisandrin B	*Schisandra chinensis*	Bcl-2, Bax and ASK1.	Myocardial infarction and infraction.	[210,211]

### 6.1 Flavonoid

**Fig. 3:**
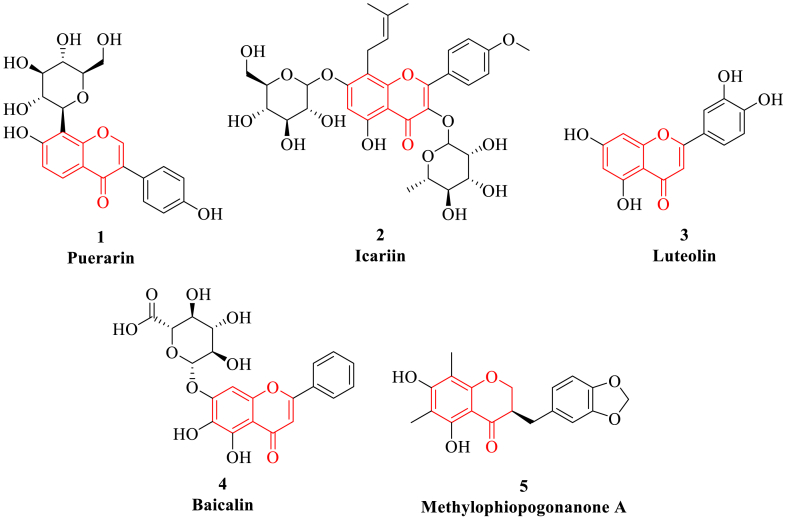
The representative flavonoids (highlighted in red). (For interpretation of the references to colour in this figure legend, the reader is referred to the Web version of this article.)

Flavonoids can pass through the blood-brain barrier (BBB) and have the functions of protecting blood vessels, dilating capillaries and dredging microcirculation. In addition, flavonoids have a variety of biological effects, such as anti-oxidation, promoting angiogenesis, anti-allergy, anti-liver injury, antitumor and endothelial cell protection, which can participate in many important biological processes including cell proliferation, differentiation, apoptosis and immune regulation through a variety of signaling pathways [97]. For example, puerarin, icariin and luteolin *etc* have the function of preventing and treating cardiovascular diseases, anti-oxidative stress, inhibiting inflammatory response, regulating organ function and other pharmacological activities (Fig. 3) [98]. Moreover, baicalin and methylophiopogonanone A *etc* can play a multi-target and synergistic role against HF by participating in biological functions including cellular energy metabolism, neuroactive ligand-receptor interactions, endovascular environmental changes and signaling pathways such as PI3K/Akt, p38MAPK and MAPK/NF-κB.

#### 6.1.1 Puerarin

Puerarin is a flavonoid glycoside extracted from the dried roots of perennial legumes *Pueraria montana* that could be used for the treatment of cardiovascular disease, cancer, diabetes and inflammation *etc* [99]. Puerarin can reduce myocardial ischemia and arrhythmia in guinea pigs and rats through inhibiting sodium ion channel (I_Na_) and L-type calcium ion channel (I_Ca-L_) and activating mitochondrial ATP-sensitive potassium channel (K_ATP_) to protect cardiomyocytes [100]. Puerarin can reduce myocardial fibrosis by increasing the activity of nuclear factor E2-associated factor 2 (Nrf2) and reducing the activity of Kelch-like epichlorohydrin-associated protein 1 (Keap1) and related downstream heme oxygenase and glutathione S-transferase [101]. Moreover, puerarin can inhibit myocardial hypertrophy through regulating extracellular signal-regulated kinase 1/2 (ERK1/2), p38MAPK and NF-κB pathways. Meanwhile, puerarin can significantly promote the formation of vascular endothelial cells and improve the microcirculation of myocardium.

#### 6.1.2 Icariin

Icariin is the main bioactive component of *Epimedium brevicornum Maxim*, which exhibits neuroprotection, bone protection, cardiovascular protection and other effects [102]. Icariin can improve left ventricular dysfunction and ventricular remodeling in congestive HF rats via inhibiting the activity of MMP 2/9, reducing collagen production and inhibiting cardiomyocyte apoptosis [103]. Icariin can significantly inhibit the apoptosis of rat cardiomyocytes caused by endoplasmic reticulum stress (ERS) inducer tunicamycin. Its mechanism of action mainly includes inhibiting the activity offers-related proteins such as glucose-regulated protein 78 (GRP78), GRP94 and C/EBP homologous protein (CHOP) and caspase-3, reducing the production of reactive oxygen species (ROS) and mitochondrial membrane potential, thus alleviating excessive ERS-caused cardiomyocyte damage [104]. In addition to inhibiting myocardial fibrosis through TGF-β1/Smad2 signaling pathway, icariin can also inhibit myocardial fibrosis and delay the progression of HF by regulating the protein expression of α-smooth muscle actin (α-SMA) and MMPs/MMP-1 [105].

#### 6.1.3 Luteolin

Luteolin is an important flavonoid that exist naturally in the form of aglycones or glycosides in *Reseda odorata*, which can be used to reduce the mortality of patients with coronary heart disease, inhibit the myocardial damage of diabetic cardiomyopathy and has a protective effect on cardiovascular [106]. Luteolin can significantly improve the cardiac function of rats with myocardial infarction, reduce the release of myocardial enzymes and inflammatory markers after myocardial infarction, upregulate autophagy and improve mitochondrial biosynthesis. Luteolin may inhibit the activation of TLR4/MyD88/NF-κB signaling pathway, reduce the levels of inflammatory factors such as IL-1β, IL-6 and TNF-α in myocardial tissue, and inhibit the inflammatory response in myocardial tissue, so as to play a protective role in HF [107,108]. Moreover, luteolin can reduce myocardial oxidative stress and enhance cardiac function, as well as promoting the expression of sarcoplasmic reticulum calcium ATPase2α (SERCA2α) and improving myocardial systolic and diastolic function [109]. In particular, luteolin can increase the levels of oxidative stress markers superoxide dismutase (SOD) and glutathione peroxidase (GSH-Px) through inhibition of superoxide generation and apoptosis of cardiomyocytes in Akt and signal transducer and activator of transcription 3 (STAT3) pathways, and inhibition of autophagy of I/R rat cardiomyocytes through mTOR pathway [110].

#### 6.1.4 Baicalin

Baicalin is one of the main active components of *Scutellaria baicalensis Georgi*, which has many effects such as scavenging oxygen free radicals, alleviating tissue I/R injury, promoting cell apoptosis and antitumor [111]. Baicalin has a good protective effect on human cardiomyocytes injured by lipopolysaccharide (LPS), which can reduce cardiomyocyte apoptosis through decreasing the expression of cytochrome C (Cyt C), apoptosis protease activating factor 1 (Apaf-1), caspase-3 and caspase-9 [112]. In addition, bacaicalin also has a protective effect on ERS-induced cardiomyocyte apoptosis through CHOP/endothelial nitric oxide synthase (eNOS)/NO pathway [113]. Baicalin can reduce cerebral edema in a dose-dependent manner, possibly by inhibiting NF-κB pathway and MMP-9 expression. Baicalin promotes the release of NO through PI3K/Akt/eNOS pathway, and alleviates the necrosis of cardiac microvascular endothelial cells (CMECs) in I/R rats by inhibiting the expression of receptor-interacting protein 1 (RIP1), RIP3 and phosphorylated mixed lineage kinase domain-like protein (p-MLKL) [114]. Moreover, baicalin can inhibit myocardial remodeling by inhibiting myocardial hypertrophy, fibrosis and apoptosis and restoring the regulatory factor PPAR β/δ [115]. However, the poor solubility of baicalin leads to low bioavailability, which is the main reason to hinder the further use in clinical treatment.

#### 6.1.5 Methylophiopogonanone A

Methylophiopogonanone A is an isoflavone compound isolated from *Ophiopogon japonicus*, which is considered to be the main active ingredient in the treatment of myocardial ischemia and thrombosis [116]. Pretreatment of methylophiopogonanone A can significantly reduce myocardial infarction size and myocardial apoptosis after I/R by 60.7 % and 56.8 %, respectively, therefore alleviating I/R injury. Mechanism studies suggest that methylophiopogonanone A can alleviate the I/R-induced apoptosis of mouse cardiomyocytes and reduce the infarct size through activating the PI3K/Akt/eNOS signaling pathway, thus playing a protective role against myocardial ischemia [117]. In addition, methylophiopogonanoe A also has a protective effect on PC12 cell damage induced by hypoxia and reoxygenation, which is related to its anti-apoptosis and anti-oxidant activities through reducing the activity of SOD and catalase [118].

### 6.2 Saponin

**Fig. 4:**
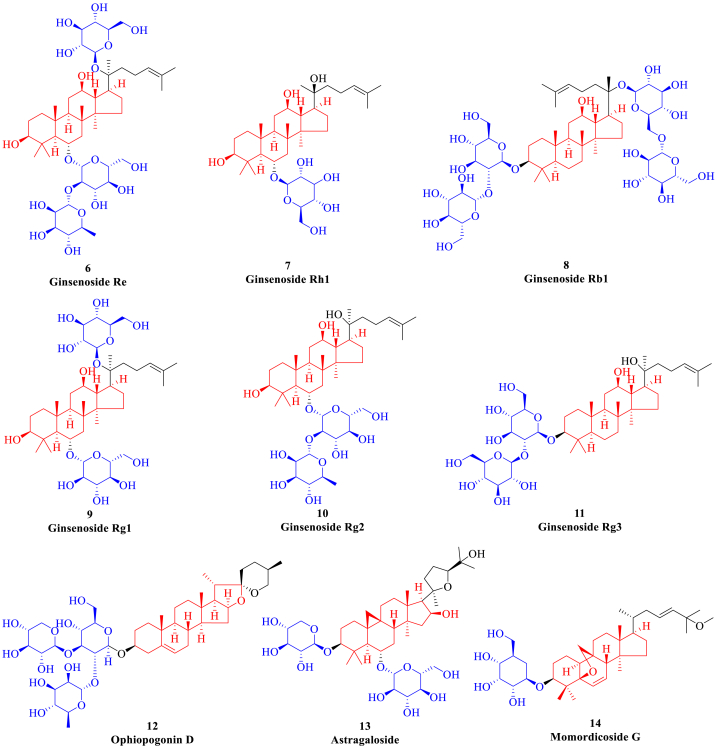
The representative saponins (highlighted in red and blue). (For interpretation of the references to colour in this figure legend, the reader is referred to the Web version of this article.)

Terpenoids are the general term for all isoprene polymers and derivatives, among which triterpenoids are widely distributed in the form of saponins and are composed of triterpenoid saponin, sugar, uronic acid and other organic substance [119]. Triterpenoid saponins include ginsenosides, astragaloside, momordicoside and ophiopogonin saponins *etc*, which have various biological activities such as antitumor, anti-inflammation, prevention and treatment of cardiovascular diseases (Fig. 4) [120]. Saponins can improve the cardiac function of HF patients and inhibit cell cardiomyocyte apoptosis, the mechanism of which may be related to the regulation of p38MAPK, Akt and NF-κB signaling pathways to reduce oxidative stress [121]. Moreover, saponins can also affect TGF-β signaling pathway by inhibiting the content of TGF-β1 in myocardial tissue and the expression of periostin protein, thereby inhibiting ventricular remodeling in HF. Finally, the mechanism of saponins in the treatment of HF is also related to the improvement of heart pumping function, intervention of ventricular remodeling and regulation of neuroendocrine system.

#### 6.2.1 Ginsenosides

Ginsenosides are the main components of *Panax ginseng* and *Panax notoginseng*. According to their chemical structures, ginsenosides can be divided into three main structural types, namely protopanaxadiol, protopanaxatriol and oleic alkane [122]. Ginsenosides can increase the left ventricular septal pacing (LVSP) and the maximum rate of rise and fall of left ventricular pressure (±dp/dt_max_), reduce the left ventricular end-diastolic pressure (LVEDP) and the content of myocardial interstitial collagen, therefore alleviating the ventricular remodeling in HF rat model [123]. The mechanism of ginsenosides improving HF may be related to the inhibition of cytoskeletal recombinant protein Rho-associated protein kinase (ROCK) and PI3K/mTOR [124]. In rats with I/R-induced myocardial injury, ginsenosides can increase the survival rate of cardiomyocytes and protect the contractile function of myocardium. The corresponding mechanism may be to reduce myocardial injury by increasing the expression of Nrf2, glutathione-cysteine ligase catalytic subunit (GCLC) and modulator subunit (GCLM) [125]. The injection of ginsenosides into rats with ischemic myocardial injury can reduce the contents of lactate dehydrogenase (LDH), creatinine kinase (CK) and myocardial hydroxyproline to improve the left ventricular function. Ginsenoside can protect myocardial function through inhibiting oxidative stress- and ERS-related enzymes apoptosis in rats with I/R-induced myocardial injury [126]. In mice with isoproterenol-induced myocardial injury, ginsenosides can increase the expression of anti-fibrotic microRNA and decrease the expression of collagen 1A1, 1A2 and 3A1, thereby reducing myocardial fibrosis [127]. Moreover, combined with paeonol, ginsenosides can inhibit the growth of myocardial fibroblasts induced by Ang-II.

#### 6.2.2 Ophiopogonin D

Ophiopogonin D, isolated from *Ophiopogon japonicus*, has widely biological activity to be used for the treatment of inflammation and cardiovascular diseases [128]. Ophiopogonin D can inhibit oxidative stress and inflammatory factors, improve the abnormal hemodynamic parameters and has a protective effect on chronic HF caused by doxorubicin [129]. The antioxidant activity of ophiopogonin D can alleviate mitochondrial membrane potential damage, improve mitochondrial productivity and balance myocardial energy homeostasis. Ophiopogonin D plays a comprehensive myocardial protective role by regulating mitochondrial autophagy activity and removing inflammatory factors [130]. Moreover, the pharmacological effect of ophiopogonin D on cardiovascular system mainly focuses on the regulation of fatty acid signaling molecules *in vivo*, such as arachidonic acid (AA), epoxyeicosatrienoic acid (EET) and hydroxyl eicosatetraenoic acid (HETE) [131].

#### 6.2.3 Astragaloside

Astragaloside is the main monomer active substance in *Astragalus membranaceus*, which is also the main index to evaluate the quality of astragalus and the material basis to exert drug effect [132]. Astragaloside has significant antioxidant activity and protective effect on cardiomyocytes injured by oxidative stress, which is mainly achieved by increasing the reserve respiratory capacity and mitochondrial ATP production [133]. After 4 weeks of continuous administration of astragaloside, the ratio of early and late filling peak velocity (E/A) and declining rate of left ventricular pressure (-dp/dt) in rats are significantly increased, the expression of protein kinase Cα (PKCα) and calcium ion sensitive receptor are increased, and the activity of SERCA2α is restored, suggesting that astragaloside can promote the recovery of cardiac diastolic function through restoring calcium homeostasis [134]. Moreover, astragaloside can not only improve cardiac function and myocardial remodeling in rats with chronic HF, increase the expression of PPAR-α and related target gene, promote the recovery of fatty acid β oxidase activity, but also improve mitochondrial function and increase the production of ATP [135]. Astragaloside can inhibit the expression of NLR family pyrin domain containing 3 (NLRP3), IL-18 and IL-6, reduce the fibrosis of primary cardiomyocytes, and thus protect the rats with LPS-induced myocardium injury. Astragaloside can also protect I/R-induced myocardial injury through inhibiting Toll-like receptor 4 (TLR4) pathway [136].

#### 6.2.4 Momordicoside G

Momordicoside G is a cucurbitane triterpenoid compound existing in the root, stem and fruit of *Momordica charantia* [137]. Momordicoside G can significantly decrease the content of metabolic end product of lipid peroxide malondialdehyde (MDA) in serum, liver and brain in mice, and increase the activities of SOD and GSH-Px [138]. Momordicoside G can inhibit the upregulation of IL-6, TNF-α, inducible nitric oxide synthase (iNOS) and cyclooxygenase-2 (COX-2) mRNA in LPS-induced mouse macrophages Raw264.7, and inhibit the activities of α-amylase and β-glucosidase, which has good anti-inflammatory effects [139]. Moreover, momordicoside G can reduce the increase of myocardial surface area induced by isoproterenol, and inhibit the upregulation of atrial natriuretic peptide (ANP), β-myosin heavy chain (β-MHC) and α-skeletal actin (α-SKA) expression of myocardial hypertrophy related embryonic genes. Momordicoside G can also alleviate cardiomyocyte hypertrophy through inhibiting the expression of glycerophospholipid metabolism related enzymes including calcium-independent phospholipase A2 (PLA2G6), diacylglycerol kinase zeta (DGKZ) and glycerophosphocholine phosphodiesterase 1 (GPCPD1) [140].

### 6.3 Phenol and phenolic acid

**Fig. 5:**
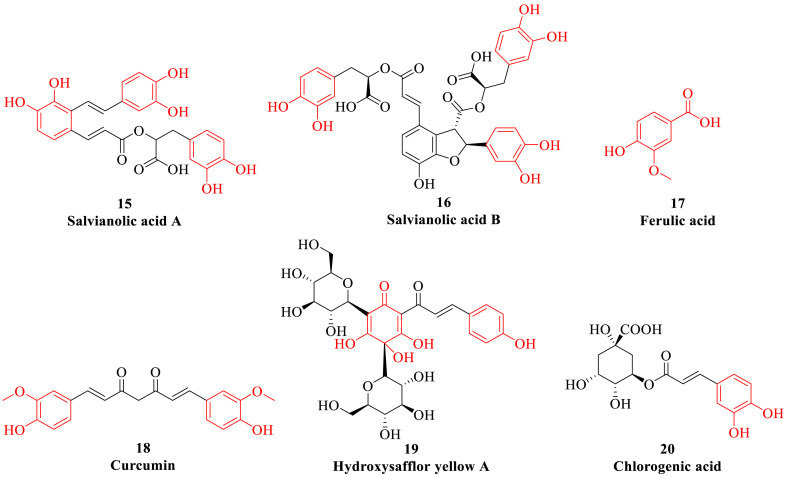
The representative phenols and phenolic acids (highlighted in red). (For interpretation of the references to colour in this figure legend, the reader is referred to the Web version of this article.)

Phenolic acids are aromatic secondary metabolites widely distributed in nature, which are also widely distributed in medicinal plants such as *Lonicera japonica Thunb*, *Taraxacum mongolicum Hand*, *Angelica sinensis* and *Ligusticum chuanxiong*. Phenolic acids are a class of metabolites containing phenolic hydroxyl group and carboxyl group, which can be divided into benzoic acid, phenylacetic acid, cinnamic acid and other phenolic acid according to their different carbon scaffolds (Fig. 5) [141]. Phenolic acids mainly exist in the form of sugar, various lipid and organic acid, which also have pharmacological effect such as anti-tumor, anti-lipid peroxidation and anti-inflammation [142]. Moreover, some representative phenols and phenolic acids such as salvianolic acid A, salvianolic acid B, ferulic acid and curcumin *etc* can be used for the treatment of myocardial fibrosis through inhibition of Ras homolog gene family member A (RhoA)/ROCK and TGF-β1/Smad2 signaling pathways. Moreover, phenolic acids can improve cardiac function and ventricular remodeling including myocardial hypertrophy, fibrosis and apoptosis in rats through regulation of oxidative stress and expression of caspase-3 and Bcl-2.

#### 6.3.1 Salvianolic acid

Salvianolic acid A, which is mainly derived from *Salvia miltiorrhiza Bunge*, is a water-soluble substance with anti-inflammatory and antioxidant effects, and can protect I/R-damaged cardiomyocytes [143]. Salvianolic acid A can decrease the expression of LDH in cardiomyocytes, inhibit apoptosis, reduce myocardial infarction size, and play a protective role in I/R injury by inhibiting the dephosphorylation of c-Jun N-terminal kinase (JNK) mediated by dual-specificity phosphatase 2 (DUSP2) and activating the phosphorylation of ERK1/2 mediated by DUSP4/16 [144]. Moreover, salvianolic acid A can enhance mitochondrial membrane stability, and increase the activity of PPAR-γ coactivator 1-α (PGC-1α) to promote mitochondrial biosynthesis, thereby alleviating cardiotoxicity induced by anti-acute lymphoblastic leukemia drugs [145]. Salvianolic acid A can also improve BNP, MDA, angiotensin and other indicators, so as to protect ischemic HF. Salvianolic acid A can inhibit myocardial fibrosis and reduce myocardial remodeling in spontaneously hypertensive rats by inhibiting the activity of MMPs, migration and differentiation of myocardial fibroblasts, intracellular adhesion factors and IL-6 secretion [146].

Salvianolic acid B can inhibit the poly(ADP-ribose)polymerase-1 (PARP-1) pathway, protect the integrity of myocardial mitochondria and nucleus, and promote the transformation of bone marrow mesenchymal stem cells into cardiomyocytes. In addition, salvianolic acid B can inhibit the phosphorylation of ERK1/2 and expression of zinc finger transcription factors GATA binding protein 4 (GATA4) and BNP, so as to significantly improve the cardiac function and myocardial remodeling in HF rats [147,148].

#### 6.3.2 Ferulic acid

Ferulic acid is the main organic acid active component of *Ligusticum chuanxiong*, which exists in both combined and free types [149]. Ferulic acid can significantly improve the cardiac function, decrease the concentration of Ang-II and endothelin-1, inhibit the expression of PKC-β and ERK1/2 in rats with abdominal aortic coarctation induced cardiac hypertrophy [150]. Other mechanism studies also suggest that the significant anti-hypertrophy effect of ferulic acid may be directly related to PKC/MAPK signaling pathway [151]. It is found that ferulic acid can play a protective role in myocardial ischemia injury rats by regulating autophagy, mostly due to dysfunction of autophagy is closely relate to a series of cardiovascular diseases such as myocardial ischemic injury. Ferulic acid can dose-dependently reduce myocardial infarction size and serum levels of LDH, creatine kinase and cardiac troponin, and improve the histological characteristics of injured myocardium. The related mechanism studies suggest that the myocardial protective effect of ferulic acid may be directly related to the activation of PI3K/Akt/mTOR signaling pathway and the recovery of autophagy [152].

#### 6.3.3 Curcumin

Curcumin is a polyphenol compound with diketone structure isolated from the rhizome of *Curcuma longa*, which has a good effect on the prevention and treatment of chronic HF, and plays a certain role in improving myocardial function and ventricular remodeling [153]. Curcumin has the effects of anti-oxidative stress, anti-inflammation, alleviating myocardial fibrosis and inhibiting myocardial apoptosis. In mice with myocardial infarction, curcumin can reduce the expression of human silencing regulatory protein 1 (SIRT1) after myocardial infarction, suggesting that SIRT1 activation may be the target of curcumin-mediated myocardial protection [154]. In the rabbit model of chronic HF, administration of curcumin for 10 weeks can increase the left ventricular ejection fraction (LVEF) and left ventricular fractional shortening (LVFS), and reduce myocardial fibrosis and myocardial hypertrophy. The mechanism of curcumin may be related to increasing the expression of negative regulatory factor Dickkopf-related protein 3 (DKK3) of cell proliferation and reducing the expression of p38, JNK and apoptosis signal-regulating kinase 1 (ASK1) [155].

#### 6.3.4 Hydroxysafflor yellow A

Hydroxysafflor yellow A is the most representative natural active component of *Carthamus tinctorius*, which has been used in the treatment of cardiovascular diseases for a long time with significant anticoagulation, antioxidant stress and angiogenesis activities [156]. Hydroxysafflor yellow A can significantly improve the hemodynamic parameters of ischemic myocardium, alleviate myocardial injury and promote angiogenesis through increasing the level of nucleolin and regulating the expression of vascular endothelial growth factor A (VEGF-A) and MMP-9 [157]. In addition, hydroxysafflor yellow A can significantly reduce the levels of LDH and caspase-3 in the I/R-injured heart, alleviate oxidative stress damage and apoptosis, and improve mitochondrial energy metabolism through activating PI3K/Akt signaling pathway, thus play a certain role in myocardial protection [158].

#### 6.3.5 Chlorogenic acid

Chlorogenic acid is an ester formed by the condensation of *trans*-cinnamic acid and quinic acid, which is widely found in plants such as *Eucommiaceae* and *Caprifoliaceae* [159]. Chlorogenic acid can reduce myocardial fibrosis through regulating TGF-β1/Smads signaling pathway and inhibiting ERS [160]. Moreover, chlorogenic acid can also decrease apoptosis of cardiomyocytes by reducing the expression level of caspase-3 and Bax and increasing the phosphorylation level of PI3K and Akt proteins in cardiomyocytes [161].

### 6.4 Alkaloid

**Fig. 6:**
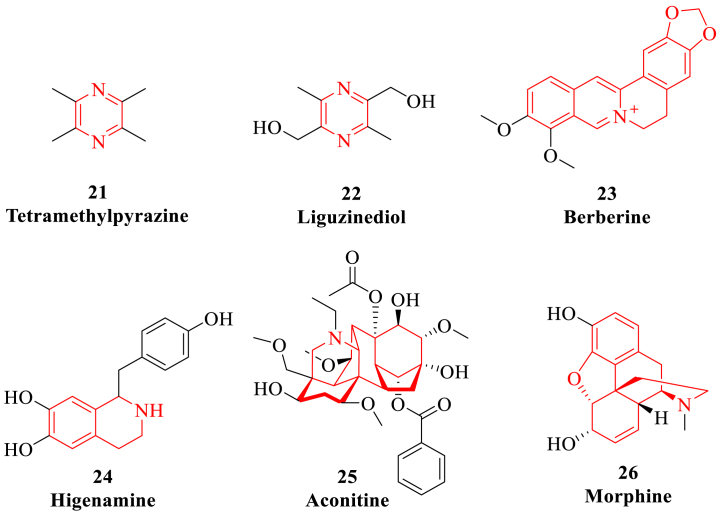
The representative alkaloids (highlighted in red). (For interpretation of the references to colour in this figure legend, the reader is referred to the Web version of this article.)

Alkaloids are a class of nitrogen-containing alkaline organic compounds, which come from a wide variety of sources and have various biological activities [162]. Alkaloids have a wide range of protective effects on cardiovascular system, such as cardiotonic effect, anti-HF, anti-shock, anti-myocardial ischemia and anti-arrhythmia [163]. For example, tetramethylpyrazine, berberine, coptisine and higenamine *etc* can exert anti-cardiomyocyte apoptosis effect to improve I/R-induced myocardial injury through regulating PI3K/Akt/eNOS signaling pathway (Fig. 6). Moreover, these promising alkaloids can also reduce ventricular remodeling and improve symptoms of HF through inhibiting the activity of RAAS. These fundamental results indicate that alkaloids are expected to be further used in clinical research of anti-oxidative damage to treat HF.

#### 6.4.1 Tetramethylpyrazine

Tetramethylpyrazine is the main alkaloid active ingredient isolated from rhizome of *Ligusticum chuanxiong.* Tetramethylpyrazine has a significant cardioprotective effect, and the synthetic tetramethylpyrazine has been a well-known drug for the treatment of cardiovascular diseases [164]. Oxygen deficiency may be one of the important pathologic mechanisms of cardiomyocyte injury in ischemic heart disease. Tetramethylpyrazine can reduce the expression of microRNA-499a and increase the activity of Sirtuin1, reverse the inactivation of PI3K/Akt pathway caused by hypoxia, and alleviate myocardial apoptosis and oxidative stress [165,166]. The oxidative stress damage caused by insufficient oxygen supply after HF has caused great damage to cardiomyocytes, which make tetramethylpyrazine play a direct role in myocardial protection by alleviating oxidative damage.

Liguzinediol is a new derivative of tetramethylpyrazine isolated from *Ligusticum chuanxiong*, which has obvious positive inotropic effect on heart [167]. Liguzinediol can reduce the cardiomyocyte damage and decrease the number of apoptosis bodies to improve the cardiac function. Moreover, liguzinediol can significantly reduce the level of Bax in HF rats and increase the level of Bcl-2 to increase the ratio of Bcl-2/Bax, which can also play a myocardial protective role and inhibit the apoptosis of cardiomyocytes by regulating the expression of apoptosis-related proteins such as Bcl-2, Bax, caspase-3 and NF-κB [168].

#### 6.4.2 Berberine

Berberine is an isoquinoline alkaloid isolated from *Coptis chinensis*, which is often used as an antibacterial drug in the treatment of gastrointestinal infections and has therapeutic effects on cardiovascular diseases [169]. Berberine can activate AMPK and PI3K/Akt/NOS signaling pathways, play an anti-apoptosis role in cardiomyocytes to improve the I/R-induced myocardial injury [170]. In addition, berberine can also reduce I/R-induced myocardial injury by regulating neurogenic locus notch homolog protein 1/hairy and enhancer of split 1-phosphatase and tensin homolog deleted on chromosome 10/Akt (Notch1/Hes1-PTEN/Akt) signaling pathway [171]. In mice with myocardial infarction, berberine can promote autophagy and reduce left ventricular remodeling and cardiac dysfunction after myocardial infarction. Its potential mechanism of action may be to enhance autophagy by inhibiting the p38MAPK signaling pathway and activating Akt phosphorylation signaling pathway [172].

#### 6.4.3 Higenamine

Higenamine is an isoquinoline alkaloid isolated from *Aconitum carmichaeli*, which has positive inotropic action, vasodilation, anti-inflammatory and antioxidant effects [173]. The combined treatment of higenamine and gingerol can improve cardiac function, reduce serum myocardial enzyme level and alleviate myocardial tissue damage. The combined therapy can significantly increase mitochondrial oxygen consumption rate and extracellular acidification rate, enhance H9c2 cell survival rate, and improve doxorubicin-induced mitochondrial dysfunction through regulating liver kinase B1 (LKB1)/AMPK/SIRT1 signaling pathway [174]. At present, higenamine has been approved by the State Food and Drug Administration (SFDA) for clinical research, which is beneficial to explore potential targets and new mechanism of action for the treatment of HF.

#### 6.4.4 Aconitine

Aconitine is the main active ingredient of *Aconitum carmichaeli*, which has a wide range of cardiovascular system protection effects such as cardiotonic effect, anti-HF, anti-myocardial ischemia and anti-arrhythmia [175]. Aconitine can increase myocardial contractility, reduce myocardial oxygen consumption and expand cardiovascular function. The mechanism may be related to the PPAR-mediated energy metabolism pathway, which enhances the oxidation capacity of fatty acids directly improves the energy supply of left ventricle through activating the PPAR-α/PGC-1α/SIRT3 signaling pathway [176,177]. Moreover, aconitine can significantly improve the neuroendocrine disorder caused by abdominal aortic coarctation, reduce ventricular remodeling and improve the symptoms of HF through inhibiting the activity of RAAS and decreasing the level of neuropoietic cytokines [178].

#### 6.4.5 Morphine

Morphine is an opioid receptor agonist, which is isolated from *Papaver somniferum* and can interfere with the normal function of various neurotransmitters in the CNS [179]. Pretreatment of morphine has a protective effect on I/R-induced myocardial injury in chronic HF rats, and the mechanism may be related to inhibiting the expression of PKCα by activating ERK and p38MAPK signaling pathways [180,181]. Moreover, pretreatment of morphine can significantly reduce the myocardial infarction size through activating the κ receptor and protect the function of sarcoplasmic reticulum in cardiomyocytes to maintain calcium homeostasis.

### 6.5 Polysaccharide

**Fig. 7:**
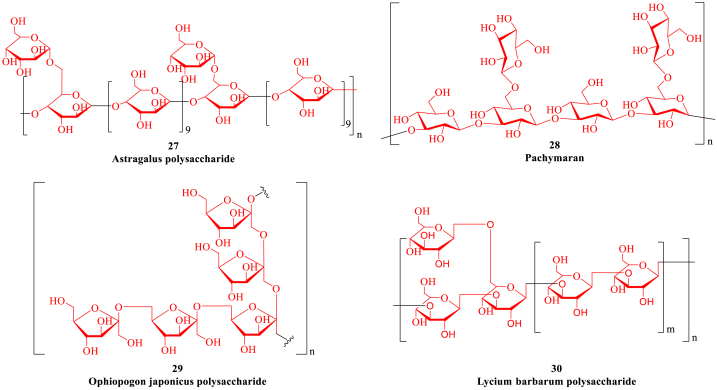
The representative polysaccharides (highlighted in red). (For interpretation of the references to colour in this figure legend, the reader is referred to the Web version of this article.)

Polysaccharide is a kind of carbohydrate substance with complex molecular structure, which is formed by condensation and dehydration of several monosaccharide molecules [182]. Plant polysaccharide have a variety of biological functions, such as immune regulation, hypoglycemic action and improvement of cardiac function (Fig. 7) [183]. For example, astragalus polysaccharide can improve cardiac function through regulating TGF-β1/PGC-1α signaling pathway. Pachymaran can work as a diuretic to play a role of myocardial protection through regulating AVP/V2R/aquaporins-2 (AQP-2) pathway. Ophiopogon japonicus polysaccharide and lycium barbarum polysaccharide can be used for the treatment of HF through regulating antioxidant enzyme level and improving microRNA-1 expression, respectively.

#### 6.5.1 Astragalus polysaccharide

Astragalus polysaccharide, as one of the main active components of *Stragalus membranaceus*, has a certain protective effect on myocardial damage through a wide range of pharmacological activities, such as correcting myocardial glucose and lipid metabolism disorder, anti-myocardial apoptosis, anti-oxidative stress, anti-myocardial fibrosis [184]. Astragalus polysaccharide can increase the level of cGMP and cyclic adenosine monophosphate (cAMP) in plasma and tissue, promote immune response, and have anti-inflammatory, antioxidant and vascular protective effects. In isoproterenol-induced myocardial hypertrophy, astragalus polysaccharide can reduce myocardial hypertrophy in model rats by regulating TNF-β/PGC-1α signaling pathway [185]. Astragalus polysaccharide can also exert its anti-myocardial hypertrophy effect through inhibiting the activities of Ca^2+^ mediated calcineurin/nuclear factor of activated T-cells 3 (NFATC3) and calcineurin kinase II (CaMK-II) [186]. In spontaneously hypertensive rats and doxorubicin-induced HF rats, astragalus polysaccharide can increase the expression of anti-apoptotic gene and anti-myocardial fibrotic protein PPAR-γ, and reduce the expression of TGF-β1, thereby improving the cardiac function [187].

#### 6.5.2 Pachymaran

Pachymaran is the main bioactive component of *Poria cocos*, which has the biological functions of antioxidant, anti-inflammatory, immunoregulation and liver protection [188]. Traditional Chinese medicine poria peel has diuretic effect, which make it be a classic diuretic in Asian traditional medicine. In recent years, it has been found that pachymaran is the main natural active ingredient for its diuretic and cardiovascular protective effects [189]. After treatment of pachymaran, the urine output of HF rats is significantly increased, and the plasma BNP level is significantly decreased. Compared with furosemide, the electrolyte disorder of rats treated with pachymaran is significantly reduced. Further mechanism studies indicate that kidney AQP-2 is significantly downregulated, the level of AVP is decreased, and mRNA expression of V2R is decreased. Therefore, it is suggested that pachymaran may play a diuretic and myocardial protective role through regulating AVP/V2R/AQP-2 pathway [190,191].

#### 6.5.3 Ophiopogon japonicus polysaccharide

Ophiopogon japonicus polysaccharide is a famous natural active compound extracted from the rhizome of *Ophiopogon japonicus* for the treatment of cardiovascular diseases [192]. Ophiopogon japonicus polysaccharide has a significant protective effect on isopropanol-induced myocardial ischemic injury in rats, and the pretreatment of it can significantly reduce ST segment elevation and cardiac index, decrease myocardial enzyme level in serum, and increase the activity of ATP synthase. In addition, pretreatment of ophiopogon japonicus polysaccharide not only increases the activities of SOD, GSH-Px and catalase in serum and myocardium, but also decreases the level of MDA [193]. Therefore, it is suggested that the mechanism of ophiopogon japonicus polysaccharide alleviating isopropanol-induced myocardial damage may be achieved by increasing endogenous antioxidant factors [194]. Moreover, ophiopogon japonicus polysaccharide has a protective effect on diabetic cardiomyopathy by regulating the level of antioxidant enzymes and improving cardiac function.

#### 6.5.4 Lycium barbarum polysaccharide

Licium barbarum polysaccharide is mainly isolated from Chinese herb *Lycium chinense*, which can significantly reduce the expression of LDH and myocardial proapoptotic gene Bax, increase the activities of Na^+^/K^+^-ATPase and Ca^2+^-ATPase, and reduce the cardiomyocyte apoptosis in I/R-injured rats [195]. In rats with HF induced by isoproterenol, administration of lyceum barbarum polysaccharide can significantly decrease the expression of a marker of myocardial injury in serum cardiac troponin (cTn-1) to improve the ventricular systolic and diastolic functions [196]. Moreover, lycium barbarum polysaccharide can inhibit the expression of microRNA-1 and improve the cardiac function and myocardial remodeling caused by microRNA-1 overexpression in rats [197].

### 6.6 Others

**Fig. 8:**
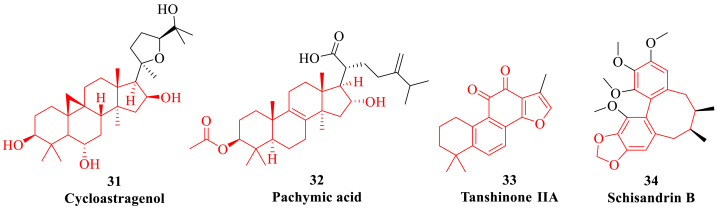
The representative natural products with anti-HF activity (scaffolds are red). (For interpretation of the references to colour in this figure legend, the reader is referred to the Web version of this article.)

In addition to the above natural compounds, some representative natural compounds with specific structures also have potential biological activities that can be employed for the treatment of HF [198]. For example, cycloastragenol and pachymic acid containing the steroid scaffold can improve the cardiac function through regulating Akt/ribosomal protein S6 kinase B1 (RPS6KB1) and ERK1/2 signaling pathways, respectively (Fig. 8). Tanshinone IIA containing the quinone scaffold can inhibit cardiomyocyte apoptosis through activating AMPK/mTOR signaling pathway and promote autophagy to improve cardiac function. Moreover, schisandrin B containing the lignan scaffold can significantly reduce the mortality after myocardial infarction. These potent natural compounds not only give the unique scaffold for the development of novel anti-HF drugs, but also provide the new mechanism and target to treat HF and related diseases.

#### 6.6.1 Cycloastragenol

Cycloastragenol is a natural compound isolated from *Astragalus membranaceus*, which has many pharmacological effects such as anti-aging, anti-inflammation, anti-fibrosis and endothelial protection [199]. In isoproterenol-induced myocardial injury model, cycloastragenol can significantly improve the abnormal cardiac hemodynamic parameters and decrease the serum levels of several neuroendocrine factors including BNP and Ang-II. Mechanism studies have shown that cycloastragenol improves the cardiac dysfunction and myocardial remodeling through inhibiting the expression of MMP-2 and MMP-9 in rats [200]. Moreover, cycloastragenol can inhibit Akt/RPS6KB1 signaling pathway, promote cardiomyocyte autophagy and then alleviate isoproterenol-induced HF in rats, suggesting that cycloastragenol may be one of the promising candidates for the treatment of congestive HF.

#### 6.6.2 Pachymic acid

Pachymic acid is a lanostane-type triterpenoid compound, which is a natural active compound extracted from *Poria cocos* with potent pharmacological effects such as anti-inflammatory, anti-oxidation and anti-apoptosis [201]. Numerous studies have shown that persistent or accumulated non-infectious myocardial inflammation is directly related to the development of HF and can be caused by a variety of injuries, including ischemia, hypoxia, hemodynamic overload and chemotherapy stimulation. Pachymic acid can reduce the mRNA expression of TNF-α, IL-1 and IL-6 induced by inflammation, inhibit myocardial apoptosis induced by LPS, and regulate the expression of apoptosis-related proteins including caspase-3, caspase-6 and caspase-9 [202]. It is suggested that the anti-inflammation and anti-apoptosis of pachymic acid may be related to the inhibition of ERK1/2 and p38MAPK signaling pathways [203,204].

#### 6.6.3 Tanshinone IIA

Tanshinone IIA is one of the representative active compounds of *Salvia miltiorrhiza*, which has significant myocardial protection in the treatment of HF and other diseases [205]. Tanshinone IIA can significantly increase the expression of autophagy related proteins such as microtubule-associated protein 1 light chain 3 (MAP1LC3), autophagy receptor sequestosome 1 (SQSTM1), lysosomal-associated membrane protein 1 (LAMP-1) and Beclin 1 to active the autophagy signaling pathway [206]. Meanwhile, tanshinone IIA can also promote autolysosome degradation and autophagosome formation to remove damaged proteins and reverse cardiomyopathy. The mTOR agonist MHY1485 can block the myocardial protective effect of tanshinone IIA, while tanshinone IIA can inhibit apoptosis and regulate the expression of key apoptotic proteins including Bcl-2, Bax, caspase-3 and caspase-7 *etc* [207]. Therefore, the mechanism studies suggest that tanshinone IIA can inhibit cardiomyocyte apoptosis through activating AMPK/mTOR signaling pathway and promote autophagy clearance, thereby protecting cardiomyocytes and improving cardiac function [208].

#### 6.6.4 Schisandrin B

In recent years, the active ingredient of *Schisandra chinensis* schisandrin B has shown potential medicinal value in the treatment of cardiovascular diseases such as hypertension and myocardial infarction [209]. Schisandrin B can play an anti-apoptotic role in cardiomyocytes through reducing the expression of proapoptotic gene Bax, decreasing the activity of ASK1 and increasing the activity of Bcl-2 gene [210]. In addition, schisandrin B can also reduce the death rate after myocardial infarction and delay the progress of myocardial remodeling after myocardial infarction to improve the cardiac function index [211].

## 7 The representative traditional Chinese medicines with anti-heart failure activity

At present, the modern medical treatment can control the symptoms and signs of HF patients with certain limitations. For example, a large number of clinical studies have found that the use of cardiotonic drugs can stimulate the systole, while the vasodilators can reduce the vascular resistance and increase the ejection fraction of the left ventricle. These treatments are effective in controlling the disease and alleviating symptoms, but there are still some problems such as large side effects, high mortality, limited long-term application and poor compliance. In the treatment of HF, TCM has the advantages that could be employed to improve the clinical symptoms, prolong the life and slow down the course of disease to improve the prognosis (Table 2) [212]. Moreover, TCM has a long history with the advantages of convenient carrying and taking in a high clinical utilization rate. According to the basis of the principle of TCM differentiation, the combined treatment with TCM will be beneficial for the treatment of HF and related diseases [213].

**Table 2:** The representative traditional Chinese medicines for the treatment of heart failure.

TCM	Composition	Functional indications	Approved
Qili Qiangxin capsule	*Astragalus membranaceus*, *Panax ginseng*, *Aconitum carmichaeli*, *Cinnamomum cassia*, *Draba nemorosa*, *Carthamus tinctorius*, *Citrus sinensis* and *Salvia mitiorrhiza.*	Tonifying qi and warming yang, promoting blood circulation and dredging collaterals, diuresis and detumescence.	Z20040141
Qishen Yiqi dropping pill	*Stragalus membranaceus*, *Salvia miltiorrhiza*, *Notoginsen radix* and *Dalbergia odorifera.*	Promoting blood circulation, relieving pain and tonifying qi.	Z20030139
Shexiang Baoxin pill	Artificial musk and bezoar, *Panax ginseng*, *Cinnamomum cassia*, *Liquidambar orientalis*, toad venom and borneol.	Dispersing fragrance and dredging collaterals, tonifying qi and strengthening heart.	Z31020068
Shenfu Qiangxin pill	*Panax ginseng*, *Aconitum carmichaeli*, *Morus alba*, *Grifola umbellata*, *Lepidium apetalum* and *Rheum palmatum.*	Tonifying qi and warming yang, strengthening heart and promoting diuresis.	Z10890018
Xinmailong injection	Xinmailong extract (complex nucleoside base and binding amino acid).	Promoting blood circulation and tonifying qi, warming yang and promoting diuresis.	Z20060443
Yixinshu capsule	*Panax ginseng*, *Schisandra chinensis*, *Ophiopogon japonicus*, *Stragalus membranaceus*, *Salvia miltiorrhiza*, *Ligusticum chuanxiong* and *Crataegus pinnatifida*.	Tonifying qi and recovering pulse, promoting blood circulation and removing blood stasis, tonifying yin and promoting saliva.	Z52020038
Wenxin granule	*Codonopsis pilosula*, *Polygonatum sibiricum*, *Radix notoginseng*, *Ambrum* and *Nardostachys jatamansi.*	Promoting blood circulation and removing blood stasis, tonifying qi and yin, fixing palpitation and restoring pulse.	Z10950026
Buyi Qiangxin tablet	*Panax ginseng*, *Stragalus membranaceus*, *Cortex periplocae*, *Salvia miltiorrhiza*, *Ophiopogon japonicus* and *Draba nemorosa*	Tonifying qi and yin, activating blood and promoting diuresis.	Z20050077

### 7.1 Qili Qiangxin capsule

On the basis of collateral disease science, Qili Qiangxin capsule is developed with the functions of promoting blood circulation to remove meridian obstruction, tonifying qi and warming yang, and inducing diuresis for removing edema [214]. The main components of Qili Qiangxin capsule are *Astragalus membranaceus*, *Panax ginseng*, *Aconitum carmichaeli*, *Cinnamomum cassia*, *Draba nemorosa*, *Carthamus tinctorius*, *Citrus sinensis* and *Salvia mitiorrhiza etc.* Qili Qiangxin capsule can improve the total effective rate and cardiac function, increase the ejection fraction, 6 min walk distance (6-MWD) and curative effect of TCM, and reduce the serum adiponectin and *N-*terminal pro-B-type natriuretic peptide (NT-proBNP) [215]. In addition, Qili Qiangxin capsule combined with conventional western medicine treatment can effectively reduce the TCM syndrome score of HF patients, reduce the number of hospitalizations aggravated by HF, and improve the quality of life and activity tolerance. Therefore, compared with the conventional treatment alone, the combined treatment of Qili Qiangxin capsule has better efficacy in improving clinical symptoms, cardiac function and NT-proBNP in HF patients. Moreover, the combined therapy containing Qili Qiangxin capsule can improve the cardiac function and hemodynamics, inhibit ventricular remodeling, reduce myocardial inflammation and effectively alleviate symptoms of HF. The results of the clinical trial demonstrated a significant increase in the proportion of patients with NT-proBNP levels ≥30 % when treated with Qili Qiangxin capsule compared to the control group, showing a difference of 16 % (47.95 % vs 31.98 %). Furthermore, it effectively reduced the incidence of cardiovascular composite endpoint events (4.51 % vs 10.93 %). During a median follow-up period of 18.3 months, Qili Qiangxin capsule demonstrated a significantly reduced incidence of major adverse cardiovascular events (MACE) compared to the placebo group (25.02 % vs 30.03 %), with a hazard ratio of 0.78. Qili Qiangxin group demonstrated a significant 24 % reduction in the risk of re-hospitalization due to worsening HF, as well as a notable 17 % decrease in the risk of cardiovascular mortality among the individual measures comprising the primary endpoint. Tangerine peel in Qili Qiangxin capsule was found to mitigate pathological myocardial hypertrophy induced by AngII and chronic HF induced by isoproterenol (ISO) through upregulation of PPAR-γ, while also reducing pathological myocardial remodeling via activation of PPAR-γ. Meanwhile, Qili Qiangxin capsule demonstrated its potential in ameliorating diabetic cardiomyopathy through the upregulation of PPAR-γ and mitigation of myocardial apoptosis induced by a hyperglycemic environment.

### 7.2 Qishen Yiqi dropping pill

Qishen Yiqi dropping pill containing *Stragalus membranaceus*, *Salvia miltiorrhiza*, *Notoginsen radix* and *Dalbergia odorifera*, which exhibit the function of promoting blood circulation, relieving pain and tonifying qi [216]. In terms of improving the clinical symptoms of chronic HF patients with qi deficiency and blood stasis syndrome, Qishen Yiqi dropping pill can improve the clinical symptoms and cardiac function of patients, protect the cardiomyocytes and inhibit the expression of inflammatory factors with good safety. Qishen Yiqi dropping pill can protect cardiomyocytes through inhibiting the apoptosis of cardiomyocytes in HF rats. Moreover, Qishen Yiqi dropping pill may increase the levels of SIRT1 and eNOS to increase the interaction between SIRT1 and eNOS, and improve the bioavailability of NO, thus stabilizing endothelial function, promoting angiogenesis, inhibiting myocardial fibrosis and apoptosis to improve cardiac function [217]. Qishen Yiqi dropping pill can also reduce the oxidative stress through increasing the level of SOD and reducing the levels of MDA, LDH and ROS, inhibit inflammation by decreasing IL-6, IL-1β and TNF-α, and inhibit cardiomyocyte apoptosis via activating Nrf2/heme oxygenase-1 (OH-1) signaling pathway. Therefore, the mechanism of Qishen Yiqi dropping pill is closely related to anti-inflammatory response, reducing myocardial fibrosis, regulating oxidative stress, promoting angiogenesis, inhibiting apoptosis of cardiomyocytes and regulating immune function. The addition of Qishen Yiqi dropping pill to standard western medicine treatment was shown in clinical studies to significantly enhance the 6-MWD of patients with HF and coronary heart disease (374.47 m vs 340.71 m). The administration of Qishen Yiqi dropping pill did not demonstrate any significant impact on composite endpoint events (including all-cause mortality and HF re-hospitalization rate) or BNP levels, however, it exhibited potential for enhancing the quality of life of patients. The administration of Qishen Yiqi dropping pill resulted in a significant improvement in the New York Heart Association (NYHA) heart function classification of patients at 30, 60, and 90 days, with effective rates of 35.41 %, 38.07 %, and 37.91 % respectively. However, both the treatment and control groups exhibited a higher LVEF post-treatment compared to pre-treatment with no difference in statistical significance (49 % *vs* 48 %). Additionally, Qishen Yiqi dropping pill was granted clinical approval for the treatment of diabetic nephropathy in 2022.

### 7.3 Shexiang Baoxin pill

Shexiang Baoxin pill consists of artificial musk and bezoar, *Panax ginseng*, *Cinnamomum cassia*, *Liquidambar orientalis*, toad venom and borneol, which also has the function of disperse fragrance and dredging collaterals, as well as tonifying qi and strengthening heart [218]. Shexiang Baoxin pill can alleviate myocardial I/R injury, and the related mechanism may be related to miR-144-3p/solute carrier family 7 member 11 (SLC7A11) signaling pathway and reducing iron death of cardiomyocytes. Shexiang Baoxin pill can improve the cardiac function, inhibit myocardial remodeling and hypertrophy through increase the expression of connexin 43 (Cx43) in HF rats. In addition, Shexiang Baoxin pill can improve the microvascular count (MVC) and microvascular density (MVD) of neovascularization, increase the microvascular length and area, and promote the proliferation, migration, adhesion and *in vitro* tube formation of vascular endothelial cells and endothelial progenitor cells [219]. Shenxiang Baoxin pill play a therapeutic role in angiogenesis, and establishes effective collateral circulation to improve cardiac ischemia and hypoxia and protect cardiac function through influencing the secretion of VEGF, hepatocyte growth factor (HGF), hypoxia-inducible factor 1 (HIF-1) and other angiogenesis regulatory factors, and regulating Notch/Delta and Hippo/YAP signaling pathways. Shexiang Baoxin pill combined with irbesartan has good clinical effect in the treatment of essential hypertension complicated with HF, which can effectively improve the cardiac function and inhibit inflammation without obviously adverse reactions. As a significant milestone in the modernization of TCM, the clinical trial of Shexiang Baoxin pill spanned over ten years and concluded successfully. The findings revealed that after two years of treatment, the combined treatment group receiving Shexiang Baoxin pill exhibited a noteworthy 26.9 % reduction in MACE incidence compared to the control group, with MACE rates at 1.9 % and 2.6 %, respectively. Furthermore, subgroup analysis revealed a significant reduction in the risk of MACE with the use of Shexiang Baoxin pill specifically in female patients and those with a body mass index (BMI) < 24 kg/m^2^, highlighting an enhanced benefit of Shexiang Baoxin pill in women and individuals with lower BMI. The incidence of MACE in patients with coronary heart disease complicated by diabetes was 4.8 % in the placebo group, whereas it decreased significantly to 2.6 % in Shexiang Baoxin pill group, representing a substantial reduction of 45.8 % compared to the placebo group. At 24 months, the incidence of secondary endpoint events in Shexiang Baoxin pill group (15.3 %) was significantly reduced by 32.3 % compared to the placebo group (22.6 %). In diabetic patients with poor glycemic control, treatment with Shexiang Baoxin pill resulted in a significant reduction of 45.5 % in the incidence of MACE (2.4 % *vs* 4.4 %), as well as a substantial decrease of 63.6 % in the occurrence of secondary endpoint events (6.0 % *vs* 16.5 %) when compared to the placebo group. The evidence-based developmental trajectory from A to E in the investigation of Shexiang Baoxin pill holds significant reference value for advancing the internationalization of evidence-based medicine in the subsequent development of TCM.

### 7.4 Shenfu Qiangxin pill

Shenfu Qiangxin pill is composed of *Panax ginseng*, *Aconitum carmichaeli*, *Morus alba*, *Grifola umbellata*, *Lepidium apetalum* and *Rheum palmatum*, which is used to tonifying qi and warm yang, and strengthen heart and promote diuresis [220]. On the basis of conventional Western medicine treatment, Shenfu Qiangxin pill can further improve the cardiac function of HF patients through inhibiting the RAAS system, regulating the levels of ANP and BNP, and reverse ventricular remodeling. Shenfu Qiangxin pill can increase LVEF in patients with chronic HF, obviously improve cardiac function and quality of life of patients through reducing the levels of BNP and Ang-II [221]. Moreover, Shenfu Qiangxin pill can significantly improve the cardiac systolic and diastolic function, reduce the TCM syndrome score and the plasma NT-proBNP level in chronic HF patients, and its mechanism may be related to inhibiting the activity of inflammatory cytokines including TNF-α and IL-6 *etc*. The clinical trial of Shenfu Qiangxin pill is currently in the research phase, with no available published data from relevant clinical trials, but only a limited number of documented clinical cases exist. For instance, Ma reported a significantly higher efficacy rate of 97 % for the combination therapy of Shenfu Qiangxin pill and ramipril, compared to the efficacy rate of 86 % observed with ramipril alone. The combined treatment of Shenfu Qiangxin pill and ramipril significantly improved the 6-MWT distance to 216.14 m, compared to the control group’s distance of 189.24 m. Li reported that the total effective rate of Shenfu Qiangxin pill combined with sacubitril valsartan sodium tablet was 95.2 %, while that of the control group was only 78.6 %. The combination treatment group exhibited a significantly higher LVEF of 47.24 %, compared to 39.40 % in the conventional group. In conclusion, the combination of Shenfu Qiangxin pill and chemical drugs demonstrated favorable efficacy and safety in the conventional management of chronic HF.

### 7.5 Xinmailong injection

Xinmailong injection is a polypeptide preparation extracted from cockroach, while the main active ingredient Xinmailong extract (complex nucleoside base and binding amino acid) of it has the effect of promoting blood circulation and tonifying qi, warming yang and promoting diuresis [222]. Xinmailong injection can decrease the level of NT-proBNP, increase LVEF and prolong 6-MWD, which indicates that Xinmailong injection can inhibit inflammatory response and promote the improvement of cardiac function and clinical symptoms [223]. Moreover, xinmailong injection can significantly inhibit the levels of pro-inflammatory factors hypersensitive C-reactive protein (hs-CRP) and IL-19, inhibit the immune inflammatory response of HF patients, while improving the microcirculation and cardiac function and reducing the cardiac preload and afterload to increase the therapeutic effect. The Phase II clinical trial of Xinmailong injection demonstrated a superior total effective rate (87.5 %–98.3 %) compared to that of the dobutamine injection control group. The Phase III clinical trial of Xinmailong injection demonstrated a total effective rate of 89 %, while the control group treated with dobutamine injection achieved an efficacy rate of 87.5 %. The Phase IV clinical study of Xinmailong injection demonstrated a total effective rate of 66.96 % in improving heart function according to NYHA criteria, and an overall effective rate of 86.96 % for TCM syndromes, surpassing that of the control group. In addition, compared to the control group, Xinmailong injection demonstrated a significant increase in the levels of 6-MWD (84 m *vs* 57 m), LVEF (3.87 *vs* 1.46), and NT-proBNP (1764 *vs* −0.21). The safety and efficacy of Xinmailong injection have been extensively demonstrated through numerous years of clinical application, garnering significant recognition from both clinicians and patients alike for its ability to enhance cardiac function and impede the progression of HF.

### 7.6 Yixinshu capsule

Yixinshu capsule, as a standardized TCM recorded in the Chinese Pharmacopoeia, is composed of seven kinds of natural products including *Panax ginseng*, *Schisandra chinensis*, *Ophiopogon japonicus*, *Stragalus membranaceus*, *Salvia miltiorrhiza*, *Ligusticum chuanxiong* and *Crataegus pinnatifida* [224]. Yixinshu capsule can significantly inhibit the cardiomyocyte damage caused by hydrogen peroxide and endothelin 1, and has a protective effect on I/R-induced myocardial ischemia. Yixinshu capsule can reduce I/R-induced myocardial injury by inhibiting mitochondria-mediated apoptosis and upregulating liver X receptor α (LXR-α) [225]. Moreover, Yixinshu capsule can promote the differentiation of bone marrow mesenchymal stem cells, so as to improve the myocardial ischemia induced HF. Yixinshu capsule can reduce the phosphorylation of RIP3 and CaMK-II, promote the phosphorylation of Ser16 residues on phospholamban (PLB), increase the expression of SERCA2α and improve the calcium uptake capacity of calcium pump, suggesting that Yixinshu capsule may regulate myocardial calcium homeostasis through the CaMK-II/PLB/SERCA2α pathway. Moreover, Yixinshu capsule can increase the expression of genes and proteins associated with calcium regulation in cardiomyocytes and decrease NCX1, thereby enhancing Ca^2+^ storage and promoting cardiac contraction. Although no clinical trials investigating the efficacy of Yixinshu capsule in HF treatment have been identified, there are several clinical case studies available regarding its usage. The combination of metoprolol and Yixinshu capsule exhibited a significantly higher overall response rate of 97.78 %, in comparison to the control group receiving metoprolol alone, which achieved an 86.67 % response rate. The combination regimen of metoprolol and Yixinshu capsule significantly augmented the LVEF to 55.21 %, whereas the group treated with metoprolol alone exhibited a lesser increase in LVEF, reaching only 50.1 %. Wang conducted a clinical study investigating the combined administration of Yixinshu capsule and spironolactone for the treatment of HF. The combination therapy exhibited a statistically significant increase in 6-MWD to 471.59 m, whereas spironolactone monotherapy resulted in an improvement of 6-MWD to 428.14 m. However, the addition of Yixinshu capsule did not significantly improve the LVEF index. The combined treatment group exhibited a LVEF value of 51.65 %, whereas spironolactone monotherapy resulted in an increased LVEF value of 55.65 %. In recent years, numerous clinical studies have demonstrated the significant potential of Yixinshu capsule in managing cardiovascular diseases such as coronary heart disease, angina pectoris, arrhythmia and HF. This is achieved through its multifaceted mechanisms including preservation of vascular endothelial function, cardiomyocyte protection, suppression of inflammatory response, regulation of calcium homeostasis and attenuation of fibrosis levels.

### 7.7 Wenxin granule

Wenxin granule contains *Codonopsis pilosula*, *Polygonatum sibiricum*, *Radix notoginseng*, *Ambrum* and *Nardostachys jatamansi*, which has the effect of promoting blood circulation and removing blood stasis, tonifying qi and yin, fixing palpitation and restoring pulse [226]. Wenxin granule can increase ejection fraction and cardiac index, decrease the levels of BNP and Ang-II, therefore improving the left ventricular systolic function in HF patients. In addition, Wenxin granule can inhibit TGF-β/Smads signaling pathway, delay the development of myocardial fibrosis, and improve the prognosis of patients with paroxysmal atrial fibrillation [227]. Wenxin granule can act on potassium, sodium and calcium plasma channels to alleviate myocardial ischemia and hypoxia through producing postrepolarization refractoriness, prolonging the effective refractory period and shortening action potential duration. Wenxin granule can also improve hemodynamics and inhibit cardiac remodeling by regulating TNF-β/JNK/p38MAPK signaling pathway and reducing collagen deposition. Moreover, Wenxin granule can play a protective role in cardiomyocytes by activating PI3K/Akt signaling pathway, decreasing the expression of Bax and caspase-3 and inhibiting the oxidative stress damage and apoptosis. In a clinical trial involving 2400 patients, Wenxin granule demonstrated a total effective rate of 83.6 % for the treatment of atrial premature beat and 83.0 % for ventricular premature beat, respectively. Additionally, it exhibited significant improvements in symptoms such as heart palpitations, chest tightness, and fatigue. The combined administration of Wenxin granule and sacubitril valsartan sodium tablet demonstrated a significantly higher total effective rate (97.78 %) in the treatment of acute myocardial infarction and HF, compared to the use of sacubitril valsartan sodium tablet alone (80.0 %). After 4 months of administration, the combined treatment group exhibited a significant increase in LVEF values to 55.75 %, while the single treatment group showed an increase to 45.02 %. These findings suggested that the addition of Wenxin granule to sacubitril valsartan sodium tablet effectively enhances the cardiac function compared to sacubactril valsartan sodium tablet monotherapy for the treatment of acute myocardial infarction and HF. The combination of Wenxin granule and trimetazidine hydrochloride achieved a total effective rate of 95 % in the treatment of coronary heart disease-related HF, whereas the monotherapy with trimetazidine hydrochloride only yielded an effective rate of 83.75 %. These findings indicated that the combination of Wenxin granule with other adjunctive pharmacotherapy exhibited enhanced efficacy in the management of HF.

### 7.8 Buyi Qiangxin tablet

Buyi Qiangxin tablet is a TCM composed of *Panax ginseng*, *Stragalus membranaceus*, *Cortex periplocae*, *Salvia miltiorrhiza*, *Ophiopogon japonicus* and *Draba nemorosa*, which can be used to treat chronic congestive HF caused by coronary heart disease and hypertensive heart disease [228]. Buyi Qiangxin tablet can activate the PI3K/Akt signaling pathway, inhibit the expression of apoptosis factors Bax and caspase-3, thus playing a myocardial protective role. Moreover, Buyi Qiangxin tablet can effectively reduce the volume load of patients with chronic HF, improve the therapeutic effect and cardiac function of patients, which is worthy of further clinical research and application. The clinical study conducted by Wu investigated the efficacy of combining Buyi Qiangxin tablet with sacubitril valsartan sodium tablet in the treatment of chronic HF. The findings revealed a significantly improved total effective rate of 95.9 % in the combined treatment group, compared to 83.7 % in the control group. After treatment four weeks, both groups exhibited a significant increase in LVEF (42.06 % *vs* 38.67 %) and 6-MWD (404.11 m *vs* 362.98 m), accompanied by a significant decrease in NT-proBNP (823.08 ng/L *vs* 987.56 ng/L). Notably, the combined treatment group demonstrated a more pronounced improvement. The clinical study conducted by Liang et al. reported a significant improvement in LVEF (48.95 % *vs* 40.45 %) and 6-MWT (430.95 m *vs* 356.23 m) when Buyi Qiangxin tablet was combined with trimetazidine dihydrochloride for the treatment of chronic HF, compared to the monotherapy group. Furthermore, in comparison to the control group receiving trimetazidine hydrochloride alone, the combined treatment group supplemented with Buyi Qiangxin tablet exhibited a more pronounced reduction in inflammatory factors such as TNF-α (11.1 pg/mL *vs* 13.3 pg/mL) and galectin-3 (16.4 μg/L *vs* 20.2 μg/L). In addition, the combination of Buyi Qiangxin tablet and other chemicals, such as l-carnitine, exhibited a favorable therapeutic effect in the management of HF by significantly enhancing cardiac function and delaying ventricular remodeling. These findings offer a novel perspective for clinical treatment.

## 8 Conclusion and prospect

HF is a group of clinical syndromes in which ventricular filling and ejection function are impaired due to various cardiac structural or functional diseases, cardiac output unmeet the needs of body tissue metabolism, and organ and tissue blood perfusion is insufficient. The common clinical HF drugs are cardiac glycoside, diuretic, vasodilator, ACEI, ARB, β receptor blocker, MRA, ARNI, SGLT-2i and others, which are used to HF prevention and disease control. However, the current therapeutic effect and prognosis of HF are not ideal, so it is of great significance to explore the potential pathogenic mechanism of HF and find new therapeutic targets and drugs.

Natural products greatly promote drug discovery, according to statistic, more than 50 % of drug molecules are closely related to natural products, which can provide pharmacophore, privileged scaffold, template molecule and modification inspiration. Moreover, natural drugs and their active ingredients have obvious advantages in stabilizing the disease, improving the cardiac function and increase the life quality that could be employed for the treatment of HF. For example, the flavonoids like puerarin, icariin, luteolin, baicalin and methylophiopogonanone A *etc* can effectively inhibit cardiomyocyte apoptosis through regulating the expression of caspase-3 and Akt/STAT3 signaling pathway, which can also be used to treat myocardial fibrosis through regulating TGF-β1/Smad2 signaling pathway. The saponins like ginsenoside Re, Rh1, Rb1, Rg1/2/3, ophiopogonin D, astragaloside and momordicoside G *etc* exhibit anti-inflammatory activity through inhibiting the expression of NF-κB, TNF-α, IL-6 and TLR4/TRIF/IRF3 signaling pathway, which can also activate autophagy and improve mitochondrial function through regulating PINK1/Parkin and Akt/mTOR signaling pathways, respectively. The phenols and phenolic acids like salvianolic acid A, salvianolic acid B, ferulic acid, curcumin, hydroxysafflor yellow A and chlorogenic acid *etc* can effectively inhibit myocardial hypertrophy and myocardial fibrosis through regulating PKC/MAPK and TGF-β1/Smads signaling pathways, respectively. The alkaloids like tetramethylpyrazine, liguzinediol, berberine, higenamine, aconitine and morphine *etc* can modulate energy metabolism and oxidative stress via regulating PPAR-α/PGC-1α/SIRT3 and PI3K/Akt/NOS signaling pathways. The polysaccharides like astragalus polysaccharide, pachymaran, ophiopogon japonicus polysaccharide and lycium barbarum polysaccharide *etc* can inhibit myocardial remodeling by inhibiting the expression of microRNA-1 and reduce myocardial hypertrophy through regulating TNF-β/PGC-1α pathway. Therefore, based on the advantages of natural products, the development of new anti-HF drugs is expected to fundamentally reduce the incidence and mortality of HF, improve the life quality and breakthrough the bottleneck of HF treatment. However, the adverse limitations like the separation and identification of active ingredients, imbalance of composition ratio and unelucidated molecular mechanism restrict the further clinical application of natural products. How to use the mechanism of natural products to discovery new targets against HF, so as to effectively apply the research results to the clinic, and develop more effective anti-HF drugs and reasonable therapeutic strategies is one of the main directions of the following research.

TCMs can improve HF by reducing myocardial fibrosis, inhibiting myocardial hypertrophy, promoting regeneration of vascular endothelial cells, improving myocardial tissue microcirculation, inhibiting apoptosis, improving myocardial remodeling and other mechanisms, which have the intervention effect on a variety of cardiovascular diseases. For example, Qili Qiangxin capsule, Qishen Yiqi dropping pill, Shexiang Baoxin pill, Shenfu Qiangxin pill, Xinmailong injection, Yixinshu capsule, Wenxin granule and Buyi Qiangxin tablet *etc* containing various potent natural products that could be used for the treatment of HF in a multi-target and multi-pathway manner. However, in the treatment of HF, the use of TCM is still in an auxiliary position. Further study on the target and mechanism of the active ingredients of TCMs against HF, optimization of the privileged structure, change of drug loading and composition ratio *etc* will provide a new direction and inspiration for the development of more safe and effective new drugs for the treatment of HF.

Since the 1990s, there has been a significant paradigm shift in the management of HF: (1) The approach has transitioned from a short-term focus on hemodynamics to a long-term remedial strategy aimed at modifying HF pathophysiology and implementing multi-mechanism/target therapy with an enhanced understanding of HF; (2) There has been a shift away from the use of cardiotonic, diuretic, and vasodilator medications towards the utilization of neuroendocrine inhibitors and other innovative therapeutic treatments including ARNI, SGLT-2i, and sGC agonists. The clinical management of HF patients primarily relies on Western medicine interventions, complemented by adjunctive Chinese medicine therapies, which serve as a supplementary approach to the treatment of HF. However, with its extensive history, rich culture, and millennia of accumulated experience, TCM undoubtedly possesses a wealth of potential in the field of anti-HF that far surpasses what has been acknowledged thus far. As a crucial source of active drug molecules, the active ingredients derived from natural products have been extensively investigated in the field of new drug development. The therapeutic effects observed in TCM prescriptions often arise from synergistic interactions among diverse active molecules present in different natural products, exhibiting characteristics of multi-target and multi-pathway actions. Nevertheless, limited separation technology and unclear mechanisms of action currently restrict their further clinical application. Therefore, by comprehensively reviewing the reported natural products and clinical drugs with anti-HF effects, we aim to provide a structural foundation for developing more innovative drug molecules while offering mechanistic insights to support the advancement of more advantageous TCM prescriptions. By delivering comprehensive, timely, and efficacious information summaries to researchers and clinicians, this review can mitigate resource wastage and redundant efforts while inspiring project design and research initiatives. Furthermore, it facilitates the advancement of natural products in HF treatment and elevates the standing of TCM within this domain.

## Funding

This work was supported by the 10.13039/501100002338Fundamental Research Funds for the Central Public Welfare Research Institutes (No. ZZ17-XRZ-057); the 10.13039/501100009620Scientific and Technological Innovation Project of China Academy of Chinese Medical Sciences (CI2021A03008 and CI2021A03001).

## Data availability statement

Data will made available on request.

## CRediT authorship contribution statement

**Xing-Juan Chen:** Writing – review & editing, Writing – original draft. **Si-Yuan Liu:** Writing – review & editing, Writing – original draft. **Si-Ming Li:** Investigation, Data curation. **Ji-Kang Feng:** Investigation, Data curation. **Ying Hu:** Investigation, Data curation. **Xiao-Zhen Cheng:** Investigation, Data curation. **Cheng-Zhi Hou:** Software, Formal analysis. **Yun Xu:** Software, Formal analysis. **Mu Hu:** Software, Formal analysis. **Ling Feng:** Supervision, Project administration, Funding acquisition, Conceptualization. **Lu Xiao:** Supervision, Project administration, Funding acquisition, Conceptualization.

## Declaration of competing interest

The authors declare that they have no known competing financial interests or personal relationships that could have appeared to influence the work reported in this paper.
